# Associations of cumulative exposure and dynamic trajectories of the combined atherogenic and frailty index with incident cardiometabolic multimorbidity: a longitudinal analysis based on the China Health and Retirement Longitudinal Study (CHARLS)

**DOI:** 10.1186/s12933-026-03235-8

**Published:** 2026-06-06

**Authors:** Xianjing Feng, Bi Deng, Yinghuan Pan, Kai Liu, Wanting Lei, Xin Tang, Jian Xia

**Affiliations:** 1 Department of Neurology, Xiangya Hospital, Central South University, Changsha, 410008 Hunan China; 2 Clinical Research Center for Cerebrovascular Disease of Hunan Province, Central South University, Changsha, Hunan China; 3 National Clinical Research Center for Geriatric Disease (Xiangya Hospital), Central South University, Changsha, Hunan China; 4 National Medical Metabolomics International Collaborative Research Center, Central South University, Changsha, China

**Keywords:** Atherogenic index of plasma, Frailty index, Cardiometabolic multimorbidity, CHARLS

## Abstract

**Background and objective:**

The atherogenic index of plasma (AIP) reflects atherogenic dyslipidemia and insulin resistance, whereas the frailty index (FI) quantifies cumulative physiological deficits across multiple organ systems. Although both the AIP and FI are independently associated with cardiometabolic multimorbidity (CMM), the joint impact of the atherogenic index of plasma-frailty index (AIPFI) on the risk of CMM remains unclear. In this study, the associations between baseline levels, cumulative AIPFI and longitudinal changes of AIPFI and the incidence of CMM were evaluated.

**Methods:**

Data were obtained from the China Health and Retirement Longitudinal Study (CHARLS). A total of 7995 participants were included in the baseline analysis, and 4,483 participants with repeated biomarker measurements in 2012 and 2015 were included in the longitudinal analysis. AIPFI was calculated via the following formula: AIPFI = AIP × FI. Multivariate Cox proportional hazards models and restricted cubic splines were applied to evaluate the associations of AIPFI with the incidence of CMM. K-means clustering characterized longitudinal AIPFI patterns. The clinical prediction model was performed in both the training and validation cohorts, as validated by receiver operating characteristic curve analysis, calibration curve analysis, and decision curve analysis. The shapley additive explanations (SHAP) method was employed to provide further explanation.

**Results:**

A total of 7995 participants were included and followed for a median of 9.0 years, during which 747 (9.3%) incident CMMs occurred. Across quartiles of the AIPFI, the risk of CMM increased progressively, with adjusted HR of 2.46 (95% CI 1.77–3.43) for Q4 compared with those for Q1. In longitudinal analyses (*n* = 4,483), participants in cluster 2, with persistently high and increasing AIPFI values, presented increased risks of CMM (HR 1.54, 95% CI 1.19–2.01). An elevated cumulative AIPFI was associated with an increased incidence of CMM (HR 1.01, 95% CI 1.01–1.01). The RCS revealed a significant positive nonlinear relationship between the baseline AIPFI and cumulative AIPFI with the risk of CMM (all *P* < 0.05, and all P for nonlinear values < 0.05). SHAP model analysis revealed hypertension, heart disease, and AIPFI as the most influential predictors.

**Conclusions:**

Both baseline and longitudinal changes in the AIPFI were independently associated with the risk of CMM. The incorporation of longitudinal monitoring of the AIPFI into routine health evaluations may enhance population-level CMM risk prediction and support more effective prevention strategies.

**Graphical abstract:**

**Figure:**
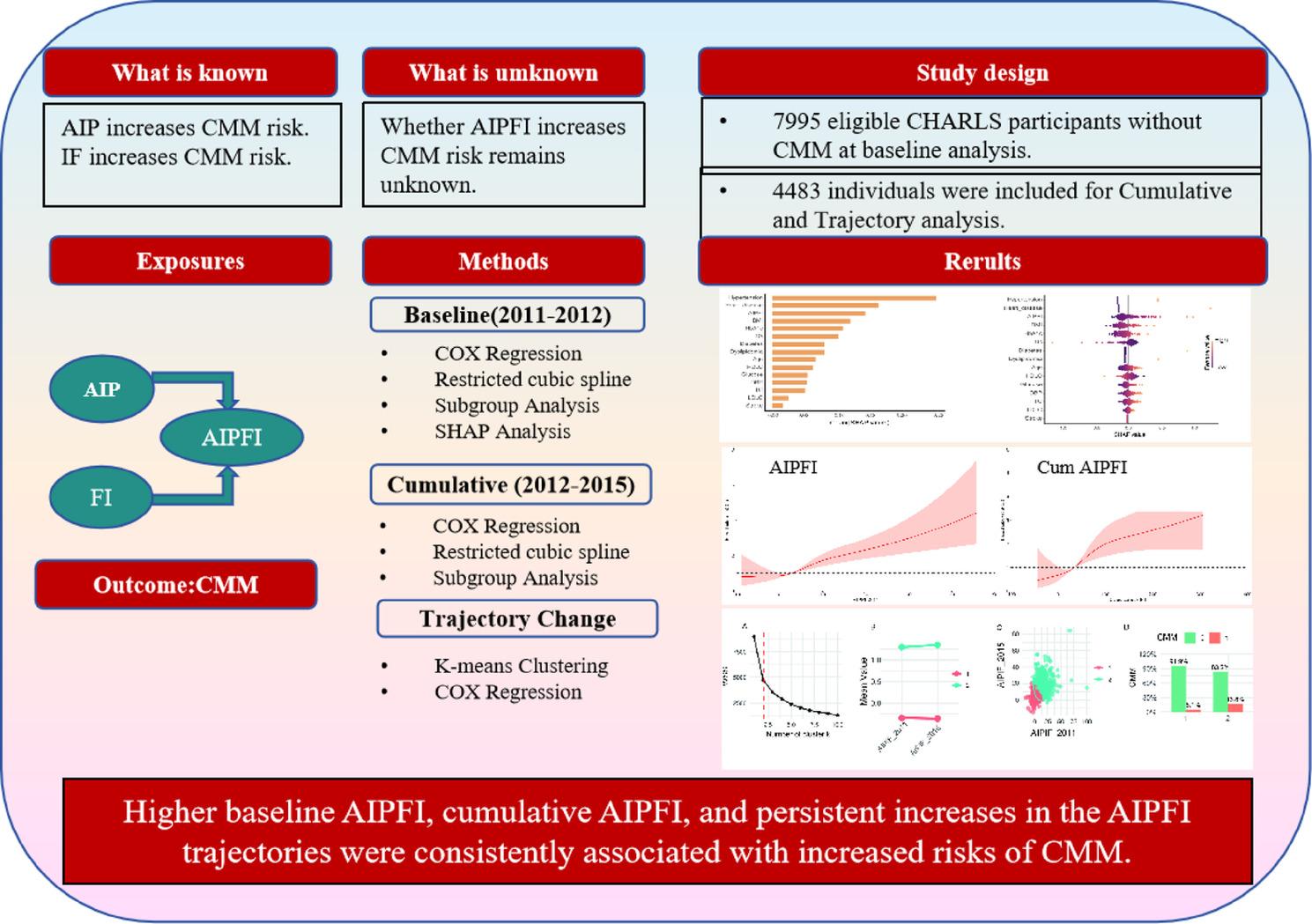

**Supplementary Information:**

The online version contains supplementary material available at 10.1186/s12933-026-03235-8.

**What is currently known about this topic?**

- Cardiometabolic multimorbidity (CMM) significantly increases mortality risk.
- The atherogenic index of plasma (AIP) indicates atherosclerosis and insulin resistance, while the frailty index (FI) reflects cumulative physiological decline.
- Although both AIP and elevated FI are established risk factors for CMM, their combined effects on CMM risk remain unclear.

**What is the key research question?**

Whether the coexistence of high levels of AIP and FI confers a more than additive risk, and whether their combination could improve early risk stratification for CMM remains unknown.

**What is new?**

Baseline AIPFI, cumulative AIPFI, and higher dynamic AIPFI cluster were associated with an increased risk of CMM.

**How might this study influence clinical practice?**

Longitudinal monitoring of AIPFI changes can help identify patients at high risk for CMM early on, supporting the recommendation for dynamic AIPFI monitoring for early CMM detection.

## Introduction

Multimorbidity, defined as the coexistence of two or more chronic conditions in an individual, represents a growing global health challenge. Among 512,723 adults aged 30–79 from the China Kadoorie Biobank, 15.8% (81,084/512,723) were found to have multimorbidity [1]. In particular, cardiometabolic multimorbidity (CMM), characterized by the co-occurrence of at least two cardiometabolic diseases (CMDs), including heart disease, stroke, and diabetes, has become increasingly prevalent in aging societies. Using data from the CHERRY study (a longitudinal cohort of 1,038,704 adults, 2010–2016), researchers observed that the prevalence of CMM rose from 2.4% to 5.9%, more than doubling over the period [2]. Moreover, CMM was associated with the greatest mortality hazard, with a hazard ratio of 2.20 [1]. Thus, early intervention targeting modifiable factors is a promising prevention strategy for CMM that could provide valuable insights into the co-prevention and co-management. Several previous observational studies have indicated that biological aging, cholesterol, triglyceride, insulin resistance-related indices, and obesity are potentially associated with CMM [3–6]. Although conventional risk factors explain part of the CMM burden, they fall short of capturing the intricate metabolic disturbances and multisystem physiological dysregulation that drive cardiovascular vulnerability. This gap has led to a growing interest in multidimensional biomarkers that can reflect broader aspects of cardiometabolic health.

The atherogenic index of plasma (AIP) is a logarithmically transformed ratio of the molar concentrations of triglycerides (TGs) to high-density lipoprotein cholesterol (HDLC) in the blood, calculated as AIP = log₁₀(TG/HDLC). It serves as a sensitive metabolic biomarker of lipoprotein profiles. The AIP is an inexpensive and easily implementable metric for stratifying cardiovascular risk. Its calculation is straightforward when standard lipid panel results are used, enhancing its translational potential. Increasing evidence supports the AIP as a predictor of metabolic disorders and atherosclerotic diseases, including diabetes [7], coronary artery disease [8], stroke [9], and CMM [10].

In parallel, the frailty index (FI), a widely used and validated measure of biological aging, quantifies health status on the basis of the accumulation of health deficits across multiple domains, including physical function, comorbidities, cognition, and psychological well-being [11]. FI offers a thorough examination of an individual’s health and has proven effective in predicting various negative health outcomes. FI has been independently associated with the risk of chronic disease [12], (e.g., cardiovascular disease (CVD) [13], stroke [14], cognitive decline [15], CMM [16]), as well as all-cause and cause-specific mortality [17].

Despite the established individual roles of the AIP and FI in predicting cardiovascular risk, they have largely been assessed in isolation. Huo et al., for the first time using the China Health and Retirement Longitudinal Study (CHARLS) database, demonstrated that a higher the combined atherogenic index of plasma and frailty index (AIPFI) was significantly associated with an increased risk of CVDs [18]. AIPFI may contribute to CVDs by accelerating vascular injury, inducing oxidative stress, and promoting atherogenesis. However, their study mainly examined the association between baseline AIPFI and CVDs. Building on their work, we conducted a more in-depth analysis of the association between AIPFI and CMM. Specifically, using the nationally representative CHARLS prospective cohort (2011–2020), which employed multistage stratified sampling and covered adults aged ≥ 45 years from both urban and rural China, we investigated the associations of baseline AIPFI, cumulative AIPFI, and longitudinal changes in AIPFI with CMM in middle-aged and older populations. Furthermore, the clinical predictive value of AIPFI for CMM remains unclear, and The shapley additive explanations (SHAP) method was used to evaluate the clinical predictive ability of AIPFI. In addition, to ensure the robustness of our findings, we conducted a series of sensitivity analyses examining the AIPFI-CMM association from different angles.

## Methods

### Study design and population

**Fig. 1:**
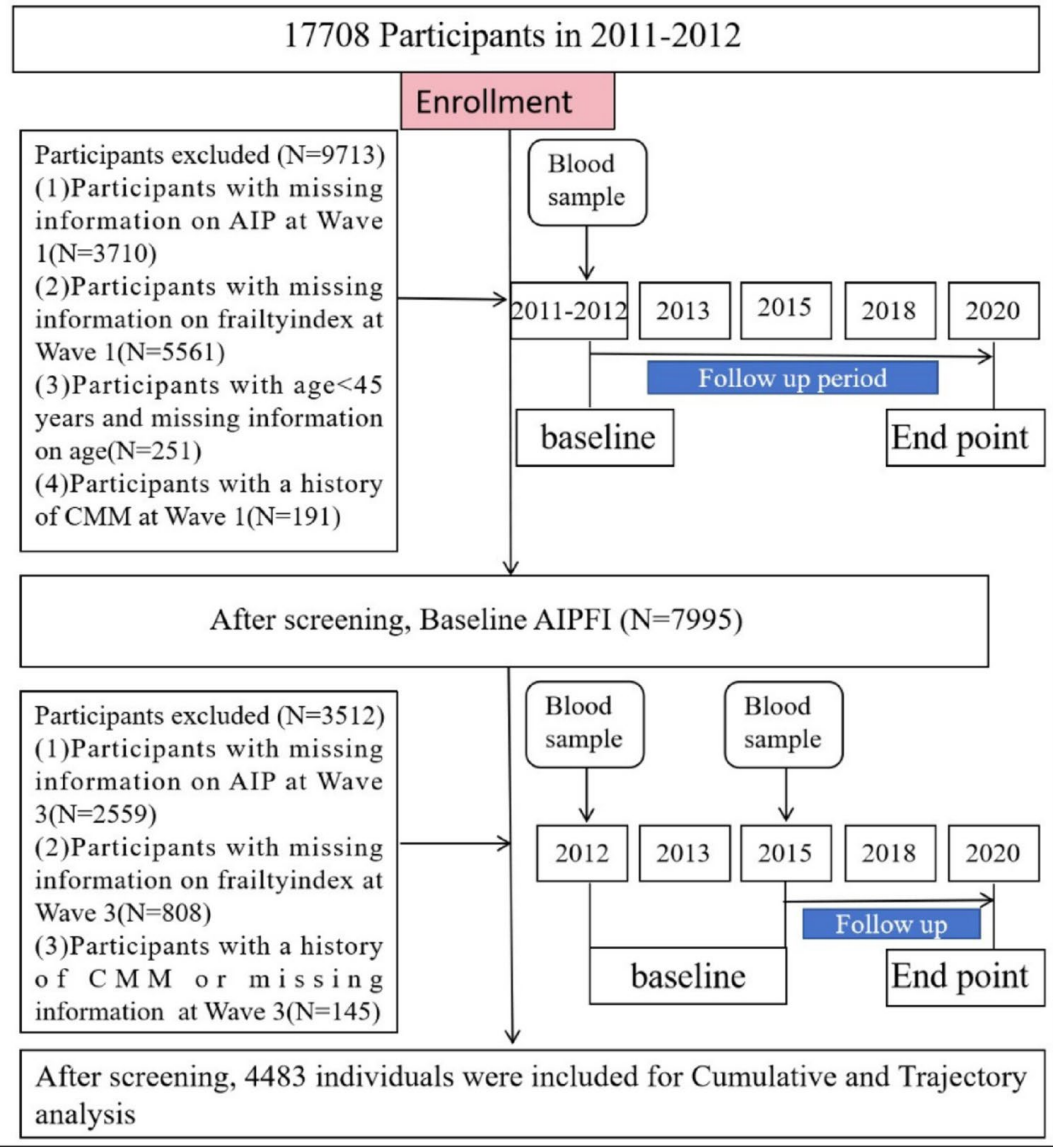
Summarizes the participant inclusion and exclusion criteria

The data were obtained from CHARLS. Baseline assessment was conducted between June 2011 and March 2012, and participants were followed up in 2013, 2015, 2018 and 2020. For our analysis, we used the baseline wave (2011–2012), and the follow-up waves in 2013, 2015, 2018 and 2020. Participants who had preexisting CMM, missing baseline biomarker data (AIP or FI), or incomplete follow-up records were excluded, resulting in a final sample of 7995 participants. The inclusion criteria allowed participants who had only one component of CMM at baseline. Table S1 compares the baseline demographic and clinical characteristics between the included participants and those excluded due to missing data. To investigate the associations between cumulative AIPFI, variations in AIPFI, and CMM risk, we further included 4,483 participants with complete AIP and FI data from 2012 to 2015 and had no history of CMM during that period. Figure 1 summarizes the participant inclusion and exclusion criteria. In total, 17,708 participants from the CHARLS cohort were included. Of these, 9713 were excluded due to incomplete CMM or AIPFI data at baseline (2011–2012), and an additional 3512 were excluded because of incomplete data in both 2011–2012 and 2015, leaving 7995 participants for the baseline analyses (2011–2020) and 4,483 for the cumulative and longitudinal analyses (2015–2020). The reporting of this study follows the STROBE guidelines for observational research [19].

### Data collection

Data collection was performed by trained interviewers via standardized questionnaires. The following baseline information was collected: gender, age, smoking, drinking, body mass index (BMI), systolic and diastolic blood pressure (SBP and DBP), hypertension, diabetes, dyslipidemia, stroke and heart disease history, fasting blood glucose (FBG), total cholesterol (TC), TG, HDLC, low-density lipoprotein cholesterol (LDLC), and hemoglobin A1c (HbA1c).

### Calculation of AIPFI, longitudinal changes of AIPFI, and cumulative AIPFI

Considering the potential synergistic effect between dyslipidemia and frailty on CMM, we constructed a composite indicator using a multiplicative model (AIPFI = AIP × FI) to capture the combined physiological burden. Following established methodology, the FI was constructed using data from the CHARLS database to quantify the accumulation of age-related health deficits. A total of 32 indicators were selected, encompassing physical function, cognitive status, and chronic conditions (Table S2). The FI was then calculated as the sum of all deficits divided by 32, multiplied by 100, yielding a score ranging from 0 to 100, where higher values indicate greater frailty [20]. The calculation formula for the AIP index was as follows: AIP = log10 (TG/HDL-C) [21]. The combined AIP and FI metric was derived via a multiplicative model: AIPFI = AIP × FI [22]. The cumulative AIPFI was calculated using data from the 2012 and 2015 waves of the CHARLS database, cumulative AIPFI (cum AIPFI) was calculated via the following algorithm: cum AIPFI = (AIPFI_2012_ + AIPFI_2015_)/2 × (2015 − 2012). The participants were subsequently divided into two groups on the basis of longitudinal changes of AIPFI via k-means analysis: Cluster 1 (the stable low-risk group) and Cluster 2 (the persistent high-risk group).

### Outcome definition and ascertainment

The primary study endpoint was incident CMM, defined as the development of at least two distinct CMDs, including diabetes, heart disease, and stroke [23, 24]. Diabetes mellitus was identified on the basis of standard laboratory and clinical criteria. The patients meeting any of the following criteria: FBG level of 126 mg/dL or greater; HbA1c level of 6.5% or higher; current use of anti-diabetic medication; or self-reported doctor diagnosis of diabetes or hyperglycemia. Heart disease (including coronary heart disease, angina, or heart failure) and stroke cases were identified through self-reported doctor’s diagnosis or documented use of cardiovascular medications. The participants were specifically asked, “Have you ever been diagnosed with heart disease?” and “Have you ever been diagnosed with a stroke?” The time-to-event was calculated as the interval from the baseline assessment to the follow-up wave in which the CMM was first identified. For participants who did not develop CMM during the study period, the follow-up time was censored at the date of their last available survey.

### Covariates

Hypertension was defined as meeting any of the following criteria: (1) self-reported physician diagnosis, (2) use of antihypertensive medications, (3) SBP ≥ 140 mmHg, or (4) DBP ≥ 90 mmHg. Dyslipidemia was identified by abnormal lipid levels (TG ≥ 150 mg/dL, TC ≥ 240 mg/dL, HDLC < 40 mg/dL, LDLC ≥ 160 mg/dL), self-reported physician diagnosis, or current use of lipid-lowering drugs. Self-reported smoking and drinking habits were also recorded (yes/no).

### Development and validation of the prediction model

The participants were randomly divided into a training cohort (70%) and a validation cohort (30%). In the training cohort, candidate predictors were those with a univariate association P value below 0.05 when comparing CMM cases to non-cases. These candidate variables were then entered into a multivariate Cox regression model to determine which were independently associated with CMM. The model was evaluated in both the training and validation cohorts using receiver operating characteristic (ROC) curve analysis, calibration curve analysis, and decision curve analysis (DCA). Model interpretability was enhanced using shapley additive explanations (SHAP) values, which reflect each feature’s contribution to the predicted outcome .

### Statistical analysis

Continuous variables with normal distributions are reported as the means ± SD, whereas nonnormally distributed variables are expressed as medians (interquartile ranges, IQRs). Categorical variables are reported as frequencies and percentages. Group comparisons were performed via χ² tests for categorical variables, ANOVA for normally distributed continuous variables, and the Kruskal-Wallis test for non-normally distributed continuous variables. The Kolmogorov‒Smirnov test was used to test the normality of the datasets, and variables with the missing data rate exceeding 20% were excluded. Quantitative data were imputed via Predictive Mean Matching (PMM), whereas categorical data were imputed via logistic regression imputation. The variables that underwent imputation included SBP, DBP, FBG, HbA1c, BMI, smoking, dyslipidemia, drinking, TC, and LDLC. The variables included in the analysis and their missing proportions are shown in Table S3.

We applied k‑means clustering (using the Euclidean distance) to identify longitudinal patterns [25]. The two available AIPFI measurements (2012 and 2015) were used as clustering inputs. To select the number of clusters, we used the elbow criterion. Each observation was assigned to the cluster with the closest mean value, which serves as the prototype of the cluster. On the basis of these data-driven assessments, the participants were classified into two distinct groups representing different longitudinal patterns of AIPFI change.

Cox proportional hazards regression models were used to investigate the associations between baseline AIPFI groups, cluster membership, cumulative AIPFI, and the incidence of CMM in the four models. The proportional hazards (PH) assumption was assessed using Schoenfeld residuals. Model 1 was unadjusted; Model 2 was adjusted for age and gender; Model 3 was adjusted for age, gender, hypertension, diabetes, dyslipidemia, heart disease, stroke, smoking, and drinking; Model 4 was adjusted for age, gender, hypertension, diabetes, dyslipidemia, heart disease, stroke, smoking, drinking, BMI, DBP, SBP, FBG, HbA1c, TC, TG, HDLC, and LDLC. Hazard ratios (HRs) with corresponding 95% confidence intervals (CIs) were calculated to quantify these associations. To address potential bias from competing mortality risks, associations were reassessed using Fine-Gray sub-distribution hazards regression, treating non-CMM-related deaths as competing events. Furthermore, restricted cubic spline (RCS) regression analysis was employed to examine the linearity and potential nonlinear relationships of baseline AIPFI and cumulative AIPFI with the risk of incident CMM. Subgroup analyses were performed after participants were stratified by age, sex, diabetes status, hypertension status, dyslipidemia status, and heart disease status. Several sensitivity analyses were also conducted to ensure the robustness of the primary findings. All the statistical analyses were conducted via R software (version 4.5.0), and a two-sided *P* value of < 0.05 was considered statistically significant.

## Results

### Population characteristics

Baseline AIPFI levels were used to categorize participants into quartiles (Table 1). Higher AIPFI quartiles were associated with older age, increased levels of BMI, SBP, DBP, FBG, HbA1c, TC, TG, LDLC, FI, and AIP, and decreased HDL-C (all *P* < 0.001). The prevalence of comorbidities (hypertension, diabetes, dyslipidemia, heart disease, stroke) and the proportions of smokers and drinkers were significantly greater in participants with higher AIPFI levels (each *P* < 0.001).

**Table 1:** participants demographics and baseline characteristics

Variables	Total (*n* = 7995)	Q1 (*n* = 1999)	Q2 (*n* = 1998)	Q3 (*n* = 1999)	Q4 (*n* = 1999)	*P*
Age, year	58.00 (51.00, 64.00)	57.00 (50.00,63.00)	57.00 (50.00,63.00)	58.00 (52.00,64.00)	59.00 (53.00,65.00)	< 0.001
Gender						< 0.001
Female	4042 (50.56)	870 (43.52)	931 (46.60)	1074 (53.73)	1167 (58.38)	
Male	3953 (49.44)	1129 (56.48)	1067 (53.40)	925 (46.27)	832 (41.62)	
Diabetes	375 (4.69)	38 (1.90)	45 (2.25)	114 (5.70)	178 (8.90)	< 0.001
Hypertension	2046 (25.59)	268 (13.41)	308 (15.42)	567 (28.36)	903 (45.17)	< 0.001
Heart disease	826 (10.33)	103 (5.15)	99 (4.95)	205 (10.26)	419 (20.96)	< 0.001
Stroke	118 (1.48)	15 (0.75)	15 (0.75)	29 (1.45)	59 (2.95)	< 0.001
Dyslipidemia	765 (9.57)	93 (4.65)	111 (5.56)	195 (9.75)	366 (18.31)	< 0.001
Drinking	3261 (40.79)	955 (47.77)	826 (41.34)	747 (37.37)	733 (36.67)	< 0.001
Smoking	3246 (40.60)	870 (43.52)	886 (44.34)	759 (37.97)	731 (36.57)	< 0.001
SBP	126.00 (113.50, 141.00)	123.00 (111.50,136.00)	125.00 (113.00,139.00)	128.00 (114.00,142.25)	129.00 (116.00,145.50)	< 0.001
DBP	74.50 (67.00, 83.00)	72.00 (65.00,81.00)	74.50 (67.00,82.50)	75.00 (67.50,83.50)	76.00 (68.50,85.00)	< 0.001
BMI	23.20 (20.93, 25.78)	22.05 (20.05,24.10)	22.93 (20.79,25.23)	23.67 (21.41,26.17)	24.45 (22.01,27.07)	< 0.001
FI	10.83 (5.36, 17.30)	7.48 (2.57,13.34)	7.37 (4.46,10.71)	10.94 (7.81,14.73)	19.75 (13.95,26.56)	< 0.001
FBG	102.06 (94.32, 112.68)	99.54 (91.98,107.46)	100.44 (93.42,109.44)	103.14 (95.22,113.76)	106.92 (97.56,122.22)	< 0.001
HbA1c	5.10 (4.90, 5.40)	5.10 (4.80,5.30)	5.10 (4.90,5.40)	5.10 (4.90,5.40)	5.20 (4.90,5.50)	< 0.001
TC	190.59 (167.40, 215.34)	185.18 (164.69,207.60)	187.89 (163.92,212.24)	192.53 (169.33,218.04)	197.55 (171.65,224.23)	< 0.001
TG	107.08 (75.22, 155.76)	62.83 (52.22,76.11)	93.81 (77.88,121.25)	124.79 (99.12,162.84)	171.69 (130.10,246.91)	< 0.001
HDL C	49.10 (40.21, 59.54)	63.02 (54.51,73.45)	51.03 (43.30,58.76)	46.39 (39.05,53.74)	40.21 (33.25,46.78)	< 0.001
LDL C	114.43 (93.17, 137.63)	109.41 (90.46,129.51)	115.98 (95.10,136.86)	118.69 (97.23,142.66)	114.82 (88.92,140.34)	< 0.001
AIP	0.33 (0.12, 0.56)	− 0.01 (− 0.10,0.08)	0.25 (0.16,0.39)	0.42 (0.29,0.58)	0.63 (0.47,0.84)	< 0.001
AIPFI	2.87 (0.72, 6.77)	− 0.05 (− 0.85,0.32)	1.68 (1.15,2.22)	4.46 (3.60,5.46)	10.82 (8.37,15.39)	< 0.001

### Association between AIPFI and incident CMM

**Fig. 2:**
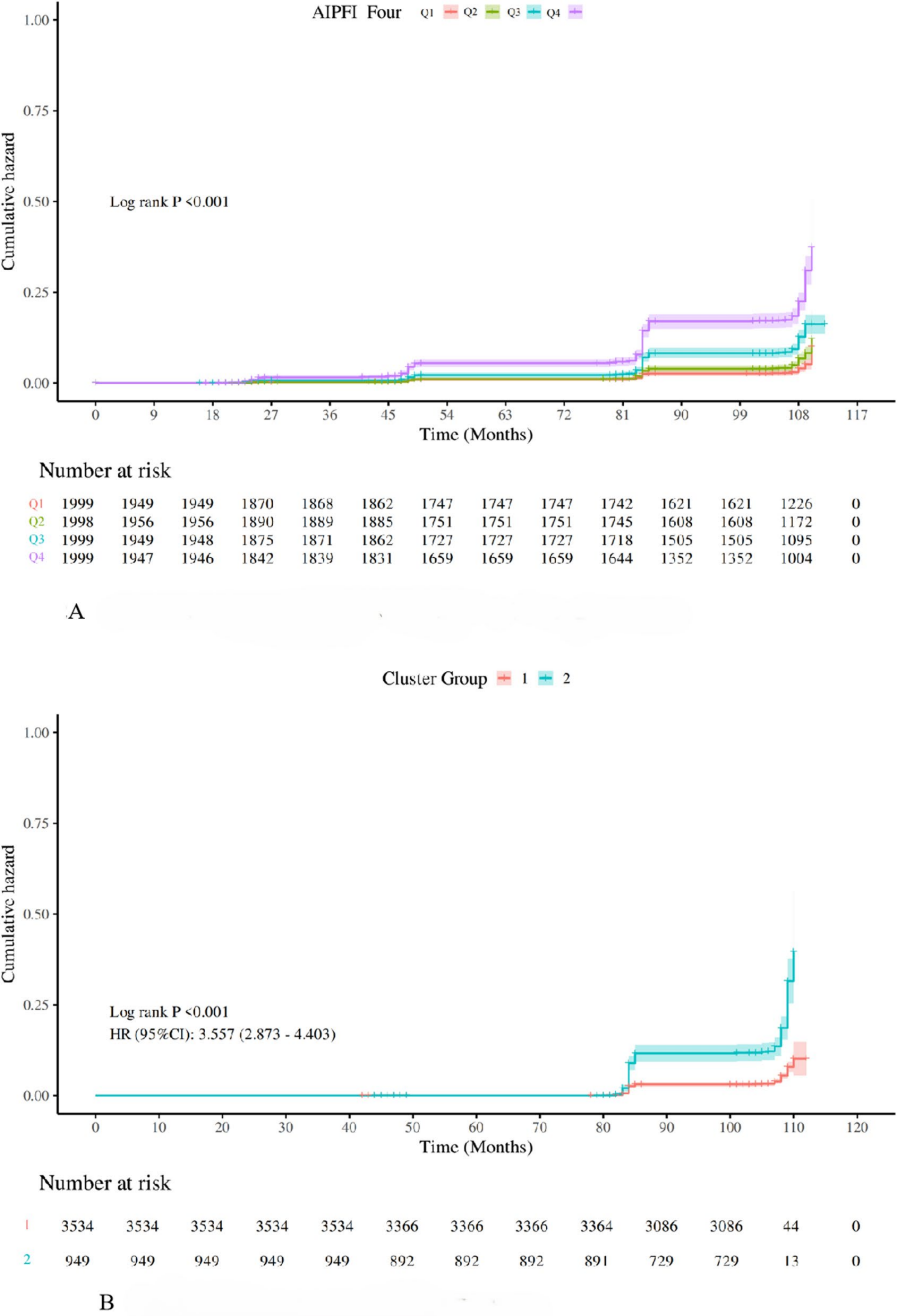
Kaplan-Meier (KM) curves for CMM by baseline AIPFI quartiles and cluster group. A KM curves for CMM by baseline AIPFI quartiles. B KM plot of CMM based on cluster group

Among the 7995 participants, 747 (9.3%) experienced CMM from 2013 to 2020 (Table S4). Figure 2A shows the Kaplan-Meier curves for CMM by AIPFI quartiles, with estimated cumulative incidence rates of 3.5%, 5.6%, 10.2%, and 18.1% for Q1 to Q4, respectively. The log-rank test revealed a significant difference in CMM incidence rates across quartiles (*P* < 0.001), indicating a graded increase in CMM risk with higher AIPFI levels.

The association between the AIPFI and CMM risk was further examined via Cox proportional hazards regression models, as shown in Table 2. The unadjusted model showed a 5.75-fold increase in CMM risk per 1‑SD increase in AIPFI (HR = 5.75, 95% CI: 4.45–7.43). The fully adjusted Model 4 indicated that the AIPFI was associated with a 2.46-fold increased risk per 1- SD increase (HR = 2.46, 95% CI: 1.77–3.43). Compared with those in Q1, the fully adjusted HRs (95% CIs) for the AIPFI in quartiles Q2, Q3, and Q4 were 1.49 (95% CI: 1.09–2.04), 2.02 (95% CI: 1.49–2.75), and 2.46 (95% CI: 1.77–3.43), respectively.

**Table 2:** Association between the AIPFI and CMM incidence (Multivariable-adjusted)

Variables	Model1		Model2		Model3		Model4	
	HR (95%CI)	*P*	HR (95%CI)	*P*	HR (95%CI)	*P*	HR (95%CI)	*P*
AIPFI	1.06 (1.06~1.07)	< 0.001	1.06 (1.05~1.07)	< 0.001	1.03 (1.02~1.04)	< 0.001	1.04 (1.02~1.05)	< 0.001
AIPFI								
Q1	1.00 (Reference)		1.00 (Reference)		1.00 (Reference)		1.00 (Reference)	
Q2	1.61 (1.19~2.17)	0.002	1.61 (1.20~2.17)	0.002	1.60 (1.18~2.16)	0.002	1.49 (1.09~2.04)	0.013
Q3	3.05 (2.33~4.01)	< 0.001	3.00 (2.28~3.93)	< 0.001	2.16 (1.64~2.84)	< 0.001	2.02 (1.49~2.75)	< 0.001
Q4	5.75 (4.45~7.43)	< 0.001	5.56 (4.30~7.20)	< 0.001	2.63 (2.00~3.44)	< 0.001	2.46 (1.77~3.43)	< 0.001

HR: Hazard Ratio, CI: Confidence Interval

Model1: Crude

Model2: Adjust: Age, gender

Model3: Adjust: Age, gender, hypertension, diabetes, dyslipidemia, heart disease, stroke, smoking, drinking

Model4: Adjust: Age, gender, hypertension, diabetes, dyslipidemia, heart disease, stroke, smoking, drinking, BMI, SBP, DBP, FBG, HbA1, TC, TG, HDLC, LDLC

### Incidence of CMM according to longitudinal changes of AIPFI and cumulative AIPFI

A total of 4483 participants had complete AIPFI data at both survey waves (2012 and 2015) and were therefore included in the longitudinal analysis of AIPFI changes and cumulative AIPFI, as presented in Table S5. During the 5.0-year follow-up period, 7.6% (339 participants) experienced CMM. The mean cumulative AIPFI was 47.68 ± 51.75. Among those without CMM (*n* = 4144), the cumulative AIPFI was 44.49 ± 48.25; whereas among those with CMM (*n* = 339), it was 86.65 ± 73.01.

**Fig. 3:**
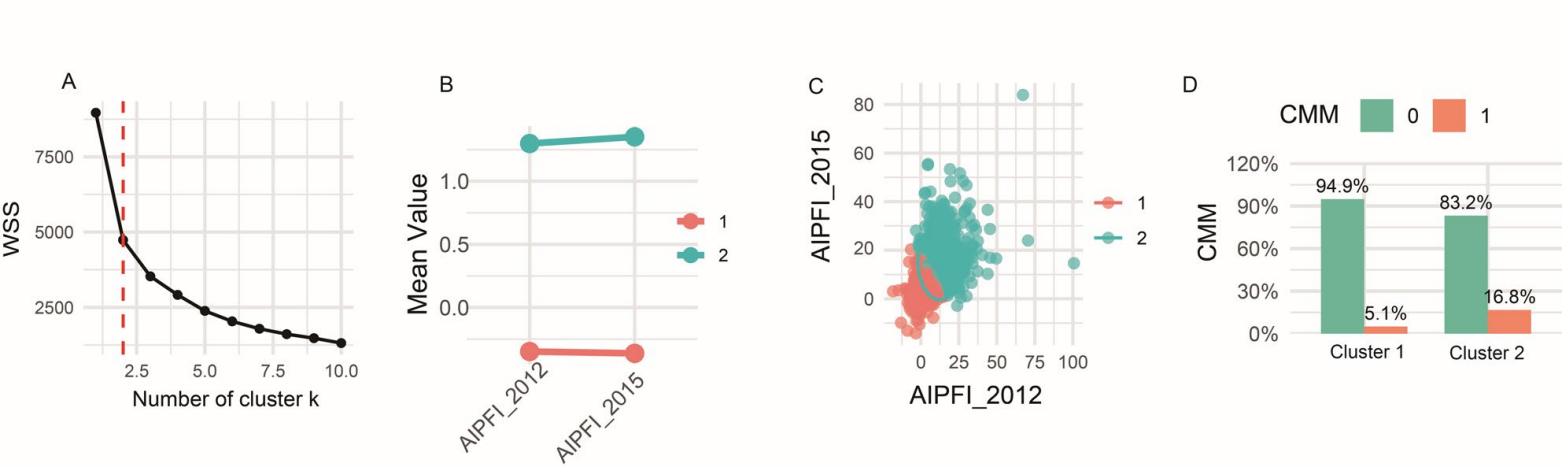
Clustering of changes of AIPFI from 2012 to 2015. Changes in AIPFI classified into two clusters using the K-means algorithm (A, B, C); Mean AIPFI value for the two clusters in 2012 and 2015 (B); Cluster 1 (Red)-Stable Low-Risk Group; Cluster 2(Green)-Persistent High-Risk Group; CMM incidence rose from 5.1% in Cluster 1 to 16.8% in Cluster 2

The K-means algorithm was used to classify AIPFI variations into two categories based on longitudinal changes of AIPFI in 2012 and 2015 (Fig. 3). In Cluster 1 (*n* = 3,534), the AIPFI varied from 1.85 (0.39–3.99) in 2012 to 3.05 (1.14–5.75) in 2015, indicating a stable low-risk group. In Cluster 2 (*n* = 949), the AIPFI varied from 11.10 (10.13–18.46) in 2012 to 13.88 (10.13–18.46) in 2015, indicating a persistent high-risk group. CMM incidence progressively increased across the two clusters (Fig. 3D), rising from 5.1% in Cluster 1 to 16.8% in Cluster 2. Table S6 presents baseline characteristics of participants stratified by AIPFI clusters. Specifically, participants in Cluster 2 had higher BMI, SBP, DBP, FBG, HbA1c, TC, and TG, and a lower HDLC (all *P* < 0.001) than those in Cluster (1) The prevalence of comorbidities (hypertension, diabetes, dyslipidemia, heart disease, stroke), and the proportions of smokers and drinkers were also significantly greater in cluster 2 than in cluster 1 (all *P* < 0.001). The log-rank test revealed a significant difference in CMM incidence between the clusters of AIPFI (*P* < 0.001) in Fig. 2B.

Table 3 summarizes the adjusted associations between the cumulative AIPFI and AIPFI longitudinal levels and CMM incidence. According to the adjusted model (Model 4), the cluster 2 group had a significantly higher CMM risk than the cluster 1 HR= 1.54 (95% CI 1.19–2.01; *P* = 0.001). Higher cumulative AIPFI (per unit increase) was significantly associated with increased CMM risk HR = 1.01 (95% CI 1.01–1.01; *P* < 0.001), and participants were further divided into four groups based on quartiles of cumulative AIPFI. Compared with the Q1 group (lowest cumulative AIPFI), the Q3 group HR = 2.48 (95% CI 1.55–3.98, *P* < 0.001) and the Q4 group HR = 2.85 (95% CI 1.72–4.72, *P* < 0.001) had a greater risk of incident CMM.

**Table 3:** Cox regression results for the association of AIPFI longitudinal change level and cumulative AIPFI with CMM (Multivariable-adjusted)

Variables	Model1		Model2		Model3		Model4	
	HR (95%CI)	*P*	HR (95%CI)	*P*	HR (95%CI)	*P*	HR (95%CI)	*P*
Cum AIPFI	1.01 (1.01~1.01)	< 0.001	1.01 (1.01~1.01)	< 0.001	1.01 (1.01~1.01)	< 0.001	1.01 (1.01~1.01)	< 0.001
*Cum AIPFI*								
Q1	1.00 (Reference)		1.00 (Reference)		1.00 (Reference)		1.00 (Reference)	
Q2	1.67 (1.02~2.72)	0.040	1.69 (1.03~2.75)	0.037	1.55 (0.95~2.53)	0.081	1.33 (0.80~2.22)	0.267
Q3	4.05 (2.63~6.25)	< 0.001	4.02 (2.61~6.21)	< 0.001	2.94 (1.89~4.56)	< 0.001	2.48 (1.55~3.98)	< 0.001
Q4	7.57 (5.01~11.43)	< 0.001	7.56 (4.99~11.46)	< 0.001	3.75 (2.42~5.81)	< 0.001	2.85 (1.72~4.72)	< 0.001
*Cluster group*								
Cluster 1	1.00 (Reference)		1.00 (Reference)		1.00 (Reference)		1.00 (Reference)	
Cluster 2	3.56 (2.87~4.40)	< 0.001	3.50 (2.81~4.35)	< 0.001	1.90 (1.50~2.42)	< 0.001	1.54 (1.19~2.01)	0.001

HR: Hazard Ratio, CI: Confidence Interval;

Model1: Crude

Model2: Adjust: Age, gender

Model3: Adjust: Age, gender, hypertension, diabetes, dyslipidemia, heart disease, stroke, smoking, drinking

Model4: Adjust: Age, gender, hypertension, diabetes, dyslipidemia, heart disease, stroke, smoking, drinking, BMI, SBP, DBP, FBG, HbA1, TC, TG, HDLC, LDLC

**Fig. 4:**
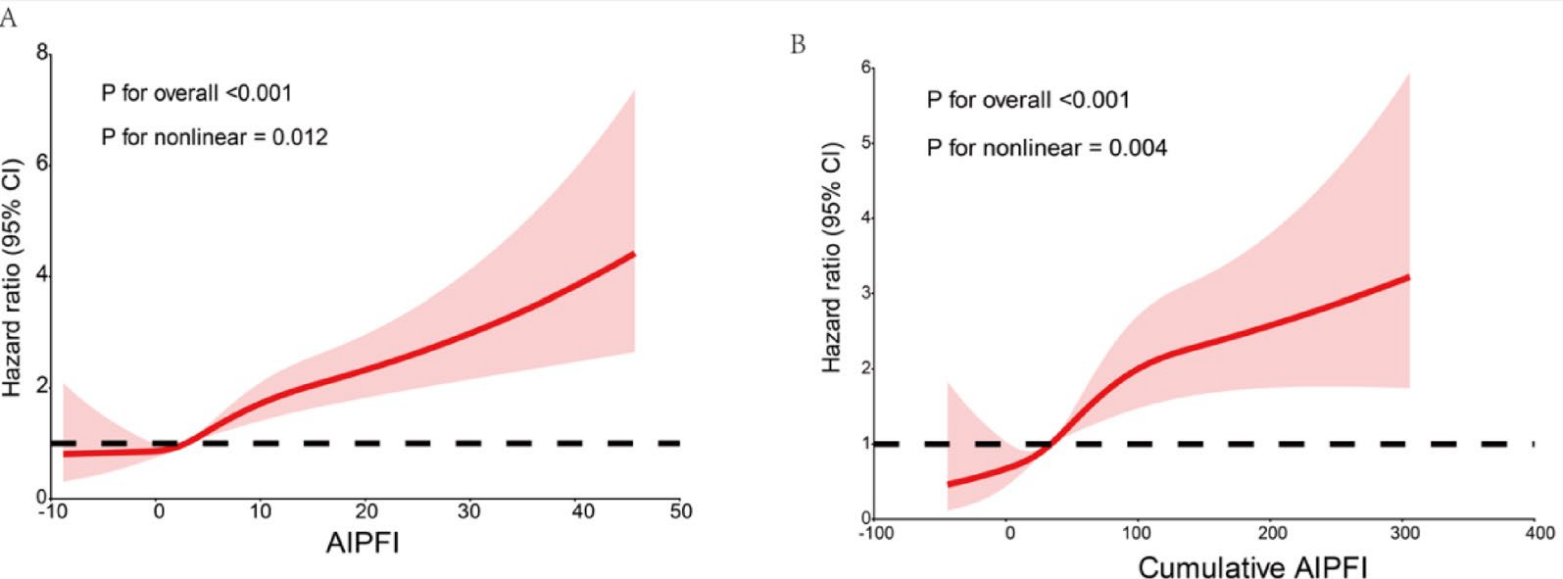
The RCS analysis of AIPFI and Cumulative AIPFI in the incidence of CMM. Adjusted for age, gender, hypertension, diabetes, dyslipidemia, heart disease, stroke, smoking, drinking, BMI, DBP, SBP, FBG, HbA1c, TC, TG, HDLC, LDLC

The RCS model analysis, adjusted for the covariates in Model 4, evaluated the associations of the baseline AIPFI and the cumulative AIPFI with incident CMM in Fig. 4. For the baseline AIPFI, the overall association with CMM was statistically significant (all *P* < 0.001), and the AIPFI displayed nonlinear relationships with incident CMM (*P* nonlinear = 0.012; Fig. 4A). Similarly, cumulative AIPFI demonstrated a strong nonlinear association with CMM (all *P* < 0.001; *P* nonlinear = 0.004; Fig. 4B).

### Subgroup analysis

**Fig. 5:**
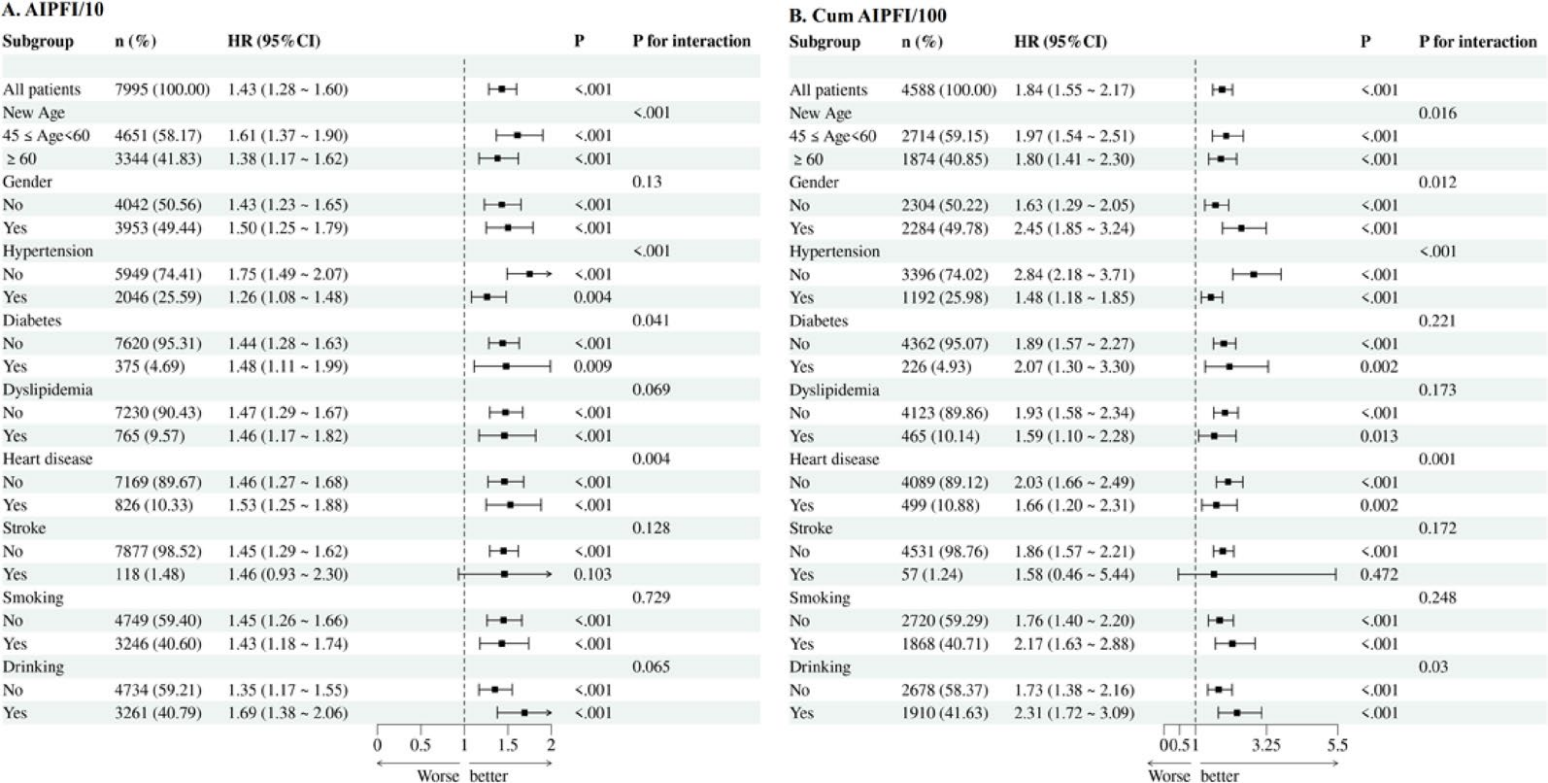
Subgroup analyses of the association of the AIPFI and cum AIPFI with the risk of CMM. Age, gender, hypertension, diabetes, dyslipidemia, heart disease, stroke, smoking, drinking, BMI, DBP, SBP, FBG, HbA1c, TC, TG, HDLC, LDLC were adjusted

Subgroup analyses (Fig. 5A and B) revealed that the baseline AIPFI and cumulative AIPFI were associated with CMM risk within certain participant subgroups. A significant interaction was found between baseline AIPFI and diabetes, hypertension, heart disease, and age (all interaction *P* values < 0.05). For cumulative AIPFI, a significant interaction was observed with gender, hypertension, and heart disease (all interaction *P* values < 0.05).

### Sensitivity analyses

We performed a series of sensitivity analyses to test the robustness of our main results.

1. In a sensitivity analysis that additionally adjusted for baseline medication history (antidiabetic, lipid‑lowering, and antihypertensive drugs) (Table S7), the association between AIPFI and CMM remained significant (e.g., Model 4: HR for Q4 = 2.51, 95% CI 1.80–3.51, *P* < 0.001).
2. In a sensitivity analysis that excluded participants receiving treatment for diabetes, hypertension, dyslipidemia, heart disease, or stroke at baseline (Table S8), and after adjusting for these medications, the association between AIPFI and CMM remained significant (e.g., Model 4: HR for Q4 = 3.42, 95% CI 2.12–5.52, *P* < 0.001).
3. After removing participants who had a baseline diagnosis of diabetes, heart disease, or stroke (Table S9), the results were generally consistent with the primary composite findings (e.g., Model 4: HR for Q4 = 3.49, 95% CI 2.25–5.42, *P* < 0.001).
4. In separate analyses, we examined the three outcomes individually: diabetes, stroke, and heart disease (Tables S10–S12). Each individual outcome showed similar associations to those observed for the composite endpoint.
5. We repeated the analysis using the 2015 survey wave as the baseline instead of the 2011–2012 wave. As shown in Table S13, AIPFI remained statistically significant in the model 4 (e.g., Model 4: HR for Q4 = 2.83, 95% CI 1.95–4.12, *P* < 0.001).
6. Standardization (Table S14): The raw AIPFI was transformed to Z‑scores to eliminate scaling effects and to make effect estimates comparable. Standardized AIPFI (Z‑score) remained significantly associated with CMM across all four models (e.g., Model4: HR = 1.27, 95% CI: 1.18–1.36, *P* < 0.001).
7. Stratification by sign (Table S14): We stratified the analysis by positive vs. non‑positive AIPFI (AIPFI > 0 vs. AIPFI ≤ 0) to assess whether the direction of the index influenced the association. In the AIPFI > 0 subgroup, each unit increase in AIPFI was associated with a 4% increased risk of CMM (Model4: HR = 1.04, *P* < 0.001). In the AIPFI ≤ 0 subgroup, the association remained statistically significant (Model4: HR = 0.79, *P* = 0.001).
8. Outlier removal (Table S14): For continuous variables with a skewed distribution, values were winsorized at the 99.9th and 0.1st percentiles to reduce the influence of extreme values. After outlier removal, the association remained robust (Model4: HR = 1.04, 95% CI: 1.03–1.05, *P* < 0.001), indicating that the results were not driven by extreme observations.
9. Reclassification of smoking/drinking (Table S15): In sensitivity analysis reclassifying smoking/drinking as “Current/Former/Never,” the positive association between AIPFI and CMM remained unchanged (Table S15). In Model 4, the HR for continuous AIPFI was 1.03 (95%CI:1.02–1.05, *P* < 0.001). For quartiles, HRs for Q2-Q4 were 1.49, 2.01, and 2.40, respectively (all *P* < 0.05), showing a clear dose-response pattern. These findings support the robustness of the main results.
10. IPW Cox Regression Analysis (Table S16): Considering the potential impact of selection bias, we conducted inverse probability weighting (IPW) to validate the robustness of our main findings. Participants were categorized into high and low AIPFI groups based on the median value, and propensity score weighting was applied. The weighted Cox regression model revealed that compared with the low AIPFI group, the high AIPFI group had a 1.72 fold higher risk of CMM (95% CI:1.33–2.23, *P* < 0.001).
11. Fine-Gray competing risks model (Table S17): we additionally conducted this model as a sensitivity analysis, with CMM as the event of interest and death as the competing event. The cumulative incidence function also indicated a higher incidence of CMM in group with AIPFI (Gray’s test, *P* < 0.001) (Fig.S1). After multiple adjustments, the competing risk model showed a graded increase in the subdistribution hazard ratio (sHR) and 95%CI by quartiles: Q2: 1.56 (95% CI: 1.14–2.13), Q3: 2.12 (95% CI: 1.56–2.89), and Q4: 2.55 (95% CI: 1.83–3.57).
12. E-value sensitivity analysis: We conducted an E-value analysis to quantify the robustness of the AIPFI-CMM association to potential unmeasured confounding. Based on the observed HR of 1.04 for AIPFI (with a 95% CI lower bound of 1.03), the E-values were calculated as follows: E-value for the point estimate = 1.244; E-value for the lower 95% CI bound = 1.206. The E-value suggested that, although the observed effect is statistically significant, it may still be affected by residual confounding.
13. Sensitivity analysis for collinearity: To test the robustness of the association between AIPFI and CMM, we performed a sensitivity analysis addressing potential collinearity. Variance inflation factor (VIF) diagnostics revealed that TG, TC, and LDL had VIF values > 5 (Table S18), indicating substantial multicollinearity. Furthermore, given that AIPFI is mathematically derived from TG and HDL-C (log [TG/HDL-C]), we additionally removed HDL-C from the model 4. Thus, in this sensitivity analysis, all conventional lipid variables (TG, TC, LDL, and HDL-C) were excluded in the model 4. As shown in the Table S19, even after completely removing traditional lipid parameters, the effect of AIPFI remained statistically significant. For the highest quartile (Q4) versus the lowest quartile (Q1), the hazard ratio was 2.20 (95% CI: 1.67–2.90, *P* < 0.001).
14. In a sensitivity analysis, we classified patients into three clusters based on longitudinal patterns of AIPFI change (Fig. S2). These clusters also showed strong predictive validity and a dose–response relationship (Table S20).
15. Model diagnostics: Schoenfeld residual tests showed PH violations for diabetes, hypertension, heart disease, and stroke (global *P* < 0.001; each *P* < 0.05; Table S21). We therefore stratified by these four variables in the final Cox model, after which no further violations were detected (global *P* > 0.05). The results of the multivariable stratified Cox regression model are shown in Table S22. The stratified model had good discrimination (C‑index = 0.803). BMI, dyslipidemia, HbA1c, and AIPFI emerged as independent risk factors (Fig. S3). Specifically, each one‑unit increase in AIPFI was associated with a 4% higher CMM risk (HR = 1.04; 95% CI: 1.03–1.05; *P* < 0.001).

### Incremental predictive value of the AIPFI

To evaluate the predictive value of the AIP, FI, and AIPFI, we performed ROC analysis (Fig. S4). The area under the curve (AUC) for the AIPFI was 0.693, compared with 0.621 for the AIP and 0.687 for the FI, indicating that the AIPFI exhibited the highest predictive value among the three biomarkers. Furthermore, adding the AIPFI to a baseline model comprising traditional risk factors (including age, sex, hypertension, dyslipidemia, and diabetes) resulted in a continuous net reclassification improvement (NRI) of 0.338 (95% CI: 0.244–0.431, *P* < 0.001) and an integrated discrimination improvement (IDI) of 0.013 (95% CI: 0.006–0.02, *P* < 0.001) (Table S23), reflecting a significant enhancement in risk reclassification and discrimination.

**Fig. 6:**
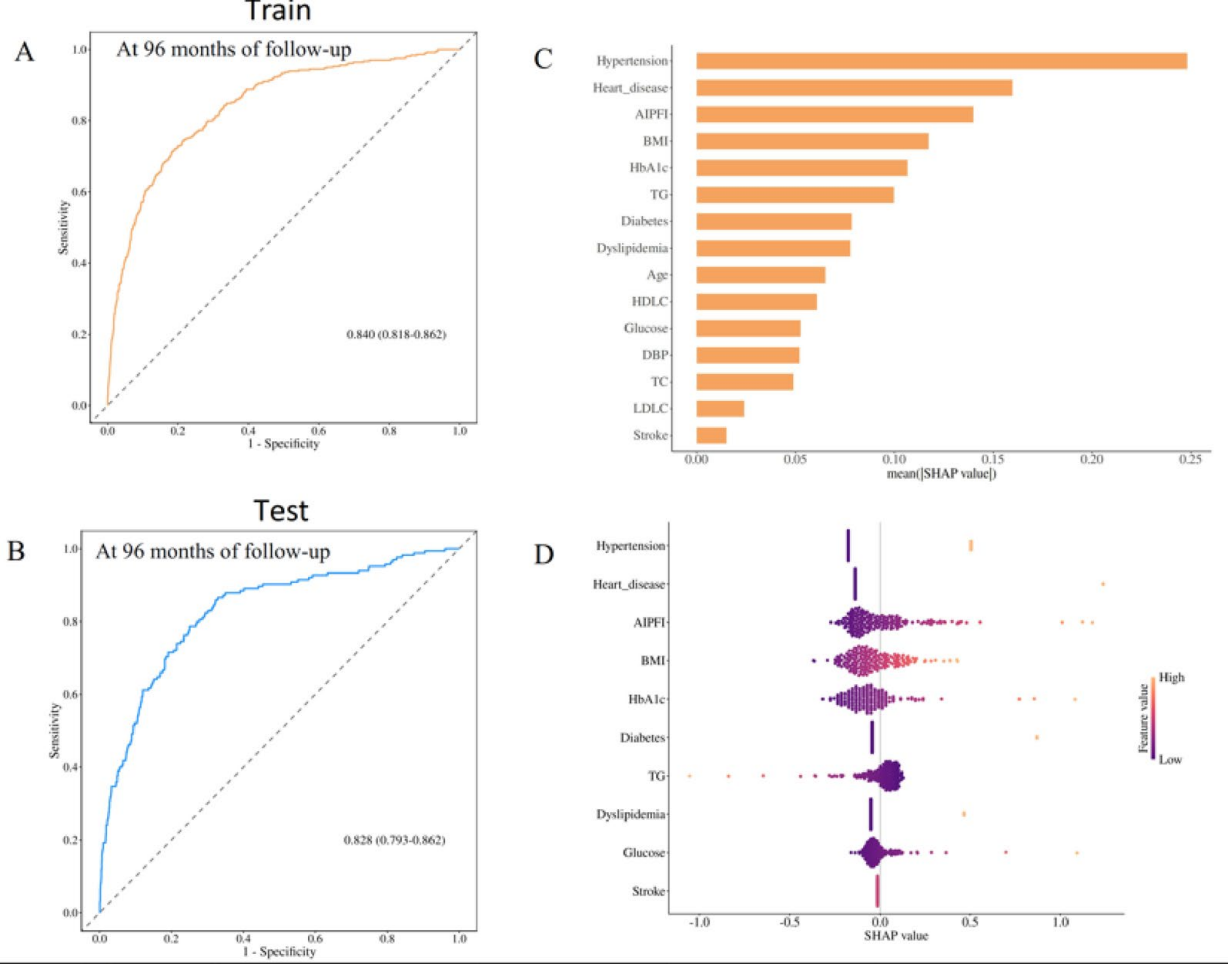
The clinical prediction analysis based on multivariate cox models, accompanied by a feature importance analysis using SHAP.The training cohort achieved an AUC of 0.84 (95% CI: 0.82–0.86) (**A**) at 96 months of follow up, whereas the validation cohort had an AUC of 0.83 (95% CI: 0.79–0.86) (**B**). Distribution of SHAP values for the ten clinical variables (**C**). The mean absolute SHAP values of ten clinical variables (**D**)

Consequently, we randomly divided 7995 participants into a 7:3 ratio, resulting in a training cohort (5,596 participants) and a validation cohort (2,399 participants) (Table S24). Seventeen variables in the training cohort with a *P* value < 0.05 comparing CMM and non-CMM groups were selected as potential predictors (Table S25). Multivariate Cox regression was subsequently performed on these variables, and ten variables were identified as independent predictors of CMM, including diabetes, hypertension, heart disease, stroke, dyslipidemia, glucose, HbA1c, BMI, TG and AIPFI (Table 4). The training cohort achieved an AUC of 0.84 (95% CI: 0.82–0.86) (Fig. 6A) at 96 months of follow up, whereas the validation cohort had an AUC of 0.83 (95% CI: 0.79–0.86) (Fig. 6B). The calibration curves was showed for both cohorts (Fig. S5A and S5B). In the training cohort, the integrated Brier score (IBS) of the Cox model was 0.0245, while that of the reference model (Kaplan-Meier null model) was 0.0213. In the validation cohort, the IBS of the Cox model was 0.0260, and that of the reference model was 0.0226. Meanwhile, the Harrell’s C-index was 0.81 in both the training and validation cohorts. Decision curve analysis revealed that the Cox model provided significant net benefits and demonstrated robust clinical validity (Fig. S5C and S5D). Additionally, the SHAP analysis was then conducted with 200 resampling iterations. The SHAP summary plot illustrates the distribution of SHAP values for the ten clinical variables, reflecting their respective impacts and contributions to the Cox regression model (Fig. 6C). Based on the mean absolute SHAP values, the three most influential factors associated with incident CMM, ranked in order of importance, were hypertension, heart disease, and the AIPFI (Fig. 6D).

**Table 4:** The results of univariate analysis and multivariate Cox regression for the incident CMM in the training cohort

Variables	Univariate analysis		Multivariate Cox regression	
	*P*	HR (95%CI)	*P*	HR (95%CI)
Age, year	< 0.001	1.02 (1.01~1.03)	0.106	1.01 (1.00~1.02)
Gender				
Female		1.00 (Reference)		1.00 (Reference)
Male	0.034	0.83 (0.70~0.99)	0.942	1.01 (0.84~1.21)
Diabetes	< 0.001	4.80 (3.81~6.05)	< 0.001	2.56 (1.88~3.48)
Hypertension	< 0.001	3.54 (2.98~4.20)	< 0.001	1.84 (1.50~2.25)
Heart disease	< 0.001	4.56 (3.78~5.51)	< 0.001	3.99 (3.22~4.93)
Stroke	< 0.001	3.06 (1.94~4.84)	< 0.001	2.88 (1.77~4.68)
Dyslipidemia	< 0.001	3.48 (2.85~4.24)	< 0.001	1.66 (1.33~2.07)
BMI	< 0.001	1.10 (1.08~1.13)	0.003	1.04 (1.01~1.06)
SBP	< 0.001	1.01 (1.01~1.02)	0.930	1.00 (0.99~1.01)
DBP	< 0.001	1.02 (1.01~1.03)	0.267	1.01 (1.00~1.02)
FBG	< 0.001	1.01 (1.01~1.01)	0.003	1.01 (1.01~1.01)
HbA1c	< 0.001	1.57 (1.49~1.66)	0.002	1.20 (1.07~1.35)
TC	< 0.001	1.01 (1.01~1.01)	0.573	1.00 (1.00~1.01)
TG	< 0.001	1.01 (1.01~1.01)	0.031	0.99 (0.99~0.99)
HDLC	< 0.001	0.98 (0.97~0.98)	0.269	0.99 (0.99~1.00)
LDLC	< 0.001	1.01 (1.01~1.01)	0.788	1.00 (0.99~1.01)
AIPFI	< 0.001	1.06 (1.05~1.07)	< 0.001	1.03 (1.02~1.04)

## Discussion

In middle‑aged and older Chinese adults, baseline and cumulative AIPFI independently predicted incident CMM, with a clear dose‑response pattern. Using k‑means clustering, persistently high AIPFI was associated with significantly greater CMM risk than persistently low AIPFI. Our study provides valuable insights into the relationships between baseline AIPFI, cumulative AIPFI, and longitudinal change of AIPFI and CMM incidence in middle-aged and older Chinese adults. The AIPFI was an independent predictor of incident CMM. Furthermore, on the basis of the K-means algorithm, our findings revealed that individuals with persistent increases in the AIPFI presented a significantly greater CMM risk than did those with persistently low AIPFI. Moreover, the cumulative AIPFI was independently associated with increased CMM risk, with participants in the higher quartiles of the cumulative AIPFI demonstrating a progressively increased CMM risk. The RCS regression model revealed a significant nonlinear relationship between baseline AIPFI and cumulative AIPFI and CMM risk. Furthermore, subgroup and sensitivity analyses confirmed the consistency of this association. Based on the mean absolute SHAP values, AIPFI was one of the three most influential factors for incident CMM. These results highlight the prognostic value of longitudinal AIPFI assessments and underscore the importance of metabolic dysregulation in CMM development.

Our findings extend previous work on the role of AIP and frailty in CVDs. The growing global burden of population aging is evident in the rising incidence and mortality of CVD. The AIP, which is positively associated with all-cause mortality in elderly patients [26], reflects IR [27], atherosclerosis [28], and cardiovascular risk [29]. Yang Zou et al. demonstrated that the cumulative AIP was correlated with prediabetes and indicated a nonlinear relationship [30]. Sijing Wu et al. reported a strong association between AIP and vulnerable plaques [28, 31]. Liu et al. and Zheng et al. reported that higher baseline and cumulative AIP values were independently associated with stroke risk in CKM syndrome stages 0–3 [32, 33]. Xiao et al. reported that six IR surrogates, including AIP, are independent predictors of CMM incidence [10]. Frailty, a risk factor for adverse health outcomes, is assessed via the frailty index, a useful tool to measure frailty progression [11]. Numerous studies have shown that the FI is an important cardiovascular risk factor. He et al. reported that worsening frailty is associated with incident CVD risk [13]. Lingli Cai et al. demonstrated that persistent frailty is associated with heart failure, coronary heart disease, and stroke risks [34]. Song et al. and Jennifer et al. reported that FI independently predicts stroke incidence and poor outcomes in older adults [35, 36]. These findings suggest a complex interplay between AIP, FI, and cardiovascular outcomes that merits further exploration.

The AIPFI, which integrates both metabolic and biological aging dimensions, has been demonstrated to be an independent risk factor for CVDs [18]. Based on the study by Huo et al. we further investigated the association between AIPFI and CMM. Analysis of data from the CHARLS study (which included 7995 participants aged ≥ 45 years at baseline and 4483 participants in the dynamic cohort) revealed that elevated baseline AIPFI, cumulative AIPFI, and persistently high AIPFI levels were consistently linked to increased CMM risk. Moreover, we performed a series of sensitivity analyses (e.g., IPW Cox regression, Fine-Gray competing risks model, standardization of AIPFI, and collinearity sensitivity analysis) to test the robustness of our main results. In the longitudinal dynamic analysis of the relationship between AIPFI and CMM, our analysis is based on two-wave repeated measurements, focusing on characterizing the inter-temporal change degree and grouping participants according to their change magnitudes. However, examining the association between long-term AIPFI trajectories and CMM risk will require additional time points. Together, these results indicate that the convergence of metabolic dysregulation and multisystem decline drives CMM susceptibility. For those already on persistently elevated trajectories, interventions must extend beyond pharmacologic control of lipids or blood pressure to include nutrition optimization, physical activity promotion, muscle preservation, cognitive engagement, and psychosocial support.

The biological mechanisms underlying the association between AIPFI and CMM in middle-aged and older people remain uncertain. However, several potential pathways have been proposed. Typical aging features include expansion of fat mass, sarcopenia, insulin resistance, and endothelial dysfunction [37, 38]. As a novel lipid marker, AIP may promote CVD by increasing insulin resistance, promoting atherosclerosis, and facilitating chronic inflammation [39]. the biological mechanisms of frailty are multifactorial and complex, ranging from DNA damage, energy metabolism, impaired vascular function, mitochondrial dysfunction, and vascular age-related arterial stiffness, many of which are further linked to CVD [40, 41]. Hui Tian et al. reported that IR indicators (including the TyG index, METS-IR, and eGDR) were significantly associated with frailty progression [42]. Shaojie Han et al. reported that inflammatory biomarkers were independently associated with a higher FI [43]. Moreover, frailty, insulin resistance, and lipid abnormalities are characterized by systemic inflammation and mitochondrial dysfunction, all of which may contribute to vascular aging and ultimately increase cardiovascular risk [44, 45]. The integration of these two indices within the AIPFI may reflect a mutually reinforcing process: metabolic derangements can fuel the inflammatory processes that drive frailty, whereas the compromised physiological state of frailty impairs the body’s ability to regulate metabolic homeostasis, thereby creating a feedforward loop that accelerates cardiometabolic deficit accumulation. Further mechanistic and prospective studies are needed to test this hypothesis.

Regarding predictive performance, the results showed that the AUC for AIPFI was 0.693, compared with 0.621 for AIP and 0.687 for FI. The modest AUC improvements may indicate an index saturation phenomenon. In the training cohort, the IBS of the Cox model was 0.0245, while that of the reference model was 0.0213. In the validation cohort, the IBS of the Cox model was 0.0260, and that of the reference model was 0.0226. Harrell’s C-index was 0.81 in both cohorts. These results indicate that although the Cox model has good discriminative ability (C-index > 0.8), its accuracy in predicting absolute risk is not superior to the null model that ignores all covariates. Future studies should examine whether AIPFI may offer added value in specific subgroups (e.g., among individuals with well-controlled traditional risk factors).

### Study strengths and limitations

Our study has several strengths. First, this study represents the first systematic evaluation of the association between AIPFI and CMM risk. Our findings highlight the potential utility of integrating AIP and FI surrogate indices into primary prevention strategies for CMM. Second, the analysis was conducted using longitudinal data from CHARLS database, providing robust evidence for the associations of AIPFI with CMM risk. Finally, the use of advanced statistical modeling techniques (e.g., K-means clustering, IPW Cox regression, the Fine-Gray competing risks model, and the SHAP model) ensured the accuracy of the results and enabled the evaluation of clinical predictive performance.

However, our study has certain limitations. First, the identification of participants with diabetes, heart disease, or stroke was primarily based on self-reported data, which may introduce misclassification bias. Future research should incorporate medical records or imaging diagnostics to improve accuracy and reduce misclassification. Second, longitudinal changes of AIPFI are based on only two repeated measurements (wave 1 and wave 3), which cannot be overinterpreted as long-term developmental patterns, this approach cannot capture the full dynamic changes of AIPFI over time. Therefore, future studies with at least three time points are needed to validate our findings and provide a more nuanced understanding of its temporal patterns. Third, the CHARLS cohort primarily included middle-aged and older Chinese individuals, and multicenter validation is needed to ensure its applicability and generalizability across diverse populations to reduce selection bias. Fourth, using the survey date as the event time introduces interval censoring, because the true onset could have occurred at any point between the previous and the current survey wave. Nevertheless, despite these limitations, the CHARLS dataset remains largely representative due to its extensive geographic coverage and substantial sample size.

## Conclusion

In this nationally representative cohort, higher baseline AIPFI, cumulative AIPFI, and persistent increases in the AIPFI trajectories were consistently associated with increased risks of CMM. These findings underscore the importance of monitoring longitudinal changes in the AIPFI and guiding the use of personalized approaches to CMM preventive strategies in clinical practice.

## Supplementary Information

Below is the link to the electronic supplementary material.

Supplementary Material 1

## Data Availability

The data supporting the findings of this study are available on the CHARLS website (http://charls.pku.edu.cn/).

## Abbreviations

AIP: Atherogenic index of plasma
AIPFI: Atherogenic index of plasma-frailty index
AUC: Area under the curve
BMI: Body mass index
CHARLS: China Health and Retirement Longitudinal Study
CI: Confidence interval
CMDs: Cardiometabolic diseases
CMM: Cardiometabolic multimorbidity
CVD: Cardiovascular disease
DBP: Diastolic blood pressure
DCA: Decision curve analysis
FBG: Fasting blood glucose
FI: Frailty index
HbA1c: Glycosylated hemoglobin
HDLC: High-density lipoprotein cholesterol
HR: Hazard ratio
IQR: Interquartile range
IR: Insulin resistance
LDLC: Low-density lipoprotein cholester
RCS: Restricted cubic splines
ROC: Receiver operating characteristic curve
SBP: Systolic blood pressure
SHAP: Shapley additive explanation
TC: Total cholesterol
TG: Triglyceride
VIF: Variance inflation factor
