# Supplementary material for: Associations of cumulative exposure and dynamic trajectories of the combined atherogenic and frailty index with incident cardiometabolic multimorbidity: a longitudinal analysis based on the China Health and Retirement Longitudinal Study (CHARLS)
Source: Cardiovasc Diabetol. 2026 Jun 6;25:228. doi: 10.1186/s12933-026-03235-8 (PMC13465527; doi:10.1186/s12933-026-03235-8)
Supplement: Supplementary file 1 [file 12933_2026_3235_MOESM1_ESM.docx]

**Supplementary Materials**

Table S1 Comparison of baseline characteristics between excluded and included populations

Table S2 The 32 items used to construct the frailty index.

Table S3 showed extracted variables and missing proportions of variables.

Table S4 Baseline characteristics of individuals classified by outcome(wave 2013 to wave 2020).

Table S5 Baseline characteristics of individuals classified by outcome (wave 2018 to wave 2020).

Table S6 Baseline characteristics of participants based on longitudinal changes in AIPFI (Cluster 1 and Cluster 2 group)

Table S7 Association between AIPFI and CMM incidence after multivariable adjustment (Covariates include medication history)

Table S8 Association between AIPFI and CMM incidence after multivariable adjustment in participants who were not receiving treatment for diabetes, hypertension, dyslipidemia, heart disease, or stroke at baseline

Table S9 Sensitivity analysis of the AIPFI on the risks of developing CMM in participants without diabetes, heart disease or stroke at baseline

Table S10 Sensitivity analysis of the AIPFI on the risks of developing diabetes.

Table S11 Sensitivity analysis of the AIPFI on the risks of developing stroke.

Table S12 Sensitivity analysis of the AIPFI on the risks of developing heart disease.

Table S13 Association between AIPFI and CMM incidence after multivariable adjustment (Using data from Wave 2015 as the baseline).

Table S14 Sensitivity analysis of the AIPFI on the risks of developing CMM (Standardization, Stratification by sign, and Outlier removal)

Table S15 Sensitivity analysis of the association between AIPFI and CMM after reclassifying smoking and drinking status as “Current/Former/Never”

Table S16 Sensitivity analysis: IPW-weighted Cox regression analysis for the association between AIPFI and CMM

Table S17 Sensitivity analysis of the relationship between AIPFI and CMM using the Fine and Gray proportional subdistribution hazards model.

Table S18 Collinearity analysis using variance inflation factor (VIF).

Table S19 Sensitivity analysis of the AIPFI on the risks of developing CMM after excluding variables with VIF >5 and TG.

Table S20 Cox regression results for the associations of longitudinal AIPFI change patterns (from 2012 to 2015 based on **three clusters**) with CMM risk.

Table S21 **Proportional Hazards Assumption Test (Schoenfeld Residuals Method)**

Table S22 Multivariable Cox regression results.

Table S23.Incremental predictive value of indicators derived from NRI and IDI

Table S24 Baseline characteristics of participants randomly divided by a 7:3 ratio into a training cohort and a validation cohort.

Table S25 Baseline characteristics of participants between the CMM and non-CMM groups into a training cohort.

**Fig.S1.** Sensitivity analysis: Cumulative incidence of CMM across AIPFI based on the Fine‑Gray competing risks model.

**Fig.S2.** Sensitivity analysis of the association between changes in AIPFI from 2012 to 2015 and CMM risk, stratified by Clusters = 3.Changes in AIPFI classified into three clusters using the K-means algorithm (A); Cluster 1 (Red)-Stable Low-Risk Group; Cluster 2(Blue)-Persistent moderate-risk group; Cluster 3 (Orange) -Persistent High-Risk Group (B); Mean AIPFI value for the three clusters in 2012 and 2015 (C).

**Fig.S3.** Multivariable Cox regression results (Forest plot of HRs).

**Fig.S4.** ROC analysis of the AIP, FI, and AIPFI. The area under the curve (AUC) for the AIPFI was 0.693, that for the AIP was 0.621, and that for the FI was 0.687. The AIPFI demonstrated the highest predictive value among the three biomarkers.

**Figure S5:** The clinical prediction analysis based on multivariate cox models.The calibration curves in both cohorts (Fig. S5A and S5B). Decision curve analysis revealed in both cohorts (Fig. S5C and S5D).

Table S1 Comparison of baseline characteristics between excluded and included populations

| Variables | Total (n = 17708) | Excluded populations  (n = 9713) | Included populations  (n = 7995) | *P* |
| --- | --- | --- | --- | --- |
|  |  |  |  |  |
| Age, year | 58.00 (50.00, 66.00) | 58.00 (50.00, 67.00) | 58.00 (51.00, 65.00) | 0.082 |
| Gender, n(%) |  |  |  | 0.216 |
| Female, n(%) | 9382 (52.98) | 5187 (53.40) | 4195 (52.47) |  |
| Male, n(%) | 8326 (47.02) | 4526 (46.60) | 3800 (47.53) |  |
| Diabetes, n(%) | 1157 (6.53) | 702 (7.23) | 455 (5.69) | <.001 |
| Hypertension, n(%) | 4645 (26.23) | 2528 (26.03) | 2117 (26.48) | 0.496 |
| Heart disease, n(%) | 2381 (13.45) | 1328 (13.67) | 1053 (13.17) | 0.330 |
| Stroke, n(%) | 567 (3.20) | 371 (3.82) | 196 (2.45) | <.001 |
| Dyslipidemia, n(%) | 1689 (9.54) | 980 (10.09) | 709 (8.87) | 0.006 |
| Drinking, n(%) | 6775 (38.26) | 3595 (37.01) | 3180 (39.77) | <.001 |
| Smoking, n(%) | 6776 (38.27) | 3717 (38.27) | 3059 (38.26) | 0.993 |
| SBP | 126.50 (113.50, 142.00) | 127.00 (113.50, 142.50) | 126.00 (113.50, 141.50) | 0.047 |
| DBP | 74.50 (67.00, 83.50) | 74.50 (67.00, 83.50) | 74.50 (67.00, 83.50) | 0.917 |
| BMI | 22.99 (20.71, 25.64) | 23.00 (20.68, 25.63) | 22.99 (20.74, 25.64) | 0.759 |
| FBG | 101.70 (93.60, 112.68) | 101.88 (93.78, 113.22) | 101.34 (93.42, 112.32) | 0.006 |
| HbA1c | 5.10 (4.90, 5.40) | 5.10 (4.90, 5.40) | 5.10 (4.90, 5.40) | 0.535 |
| TC | 188.66 (167.40, 211.47) | 188.66 (167.78, 211.08) | 188.66 (166.62, 211.86) | 0.771 |
| TG | 105.32 (75.22, 149.57) | 104.43 (75.22, 146.91) | 106.20 (76.11, 152.22) | 0.006 |
| HDL C | 49.48 (39.82, 59.54) | 49.10 (39.82, 59.92) | 49.48 (40.21, 59.54) | 0.772 |
| LDL C | 113.66 (94.33, 134.15) | 114.05 (95.49, 134.15) | 112.89 (93.17, 133.76) | 0.006 |

| Table S2 The 32 items used to construct the frailty index | | |
| --- | --- | --- |
| **No** | **Description of the items** | **Cut-off value** |
| 1 | Self-reported physician diagnosed hypertension | Yes = 1, No = 0 |
| 2 | Self-reported physician diagnosed diabetes | Yes = 1, No = 0 |
| 3 | Self-reported physician diagnosed heart disease | Yes = 1, No = 0 |
| 4 | Self-reported physician diagnosed stroke | Yes = 1, No = 0 |
| 5 | Self-reported physician diagnosed cancer | Yes = 1, No = 0 |
| 6 | Self-reported physician diagnosed arthritis | Yes = 1, No = 0 |
| 7 | Self-reported physician diagnosed chronic lung disease | Yes = 1, No = 0 |
| 8 | Self-reported physician diagnosed asthma | Yes = 1, No = 0 |
| 9 | Self-reported physician diagnosed any emotional, nervous, or psychiatric problems | Yes = 1, No = 0 |
| 10 | Self-reported physician diagnosed memory-related disease | Yes = 1, No = 0 |
| 11 | Self-reported vision problems | Yes = 1, No = 0 |
| 12 | Self-reported hearing problems | Yes = 1, No = 0 |
| 13 | Self-reported general health status | Poor or fair = 1, excellent, very good, or good = 0 |
| 14 | Difficulty with dressing | Yes = 1, No = 0 |
| 15 | Difficulty with bathing or showering | Yes = 1, No = 0 |
| 16 | Difficulty with eating | Yes = 1, No = 0 |
| 17 | Difficulty with getting in and out of bed | Yes = 1, No = 0 |
| 18 | Difficulty with using the toilet | Yes = 1, No = 0 |
| 19 | Difficulty with managing money | Yes = 1, No = 0 |
| 20 | Difficulty with taking medications | Yes = 1, No = 0 |
| 21 | Difficulty with shopping for groceries | Yes = 1, No = 0 |
| 22 | Difficulty with preparing meals | Yes = 1, No = 0 |
| 23 | Difficulty with doing housework | Yes = 1, No = 0 |
| 24 | Mobility: difficulty with walking 100 yards | Yes = 1, No = 0 |
| 25 | Mobility: difficulty with getting up from a chair after sitting for long periods | Yes = 1, No = 0 |
| 26 | Mobility: difficulty with climbing several flights of stairs without resting | Yes = 1, No = 0 |
| 27 | Mobility: difficulty with lifting or carrying weights over 10 pounds/jins | Yes = 1, No = 0 |
| 28 | Mobility: difficulty with picking up a coin from the table | Yes = 1, No = 0 |
| 29 | Mobility: difficulty with stooping, kneeling, or crouching | Yes = 1, No = 0 |
| 30 | Mobility: difficulty with reaching arms above shoulder level | Yes = 1, No = 0 |
| 31 | Depression: CESD-10 questionnaire | CESD-10 ≤10 =0 >10 =1 |
| 32 | Cognition: (memory test score + orientation test score) **/** 14 | Continuous, ranging from 0 to 1 |
| Depression is evaluated using the Center for Epidemiologic Studies Depression Scale (CESD). In the CHARLS, CESD-10 is used, and the total score ranges from 0 to 30. The memory score is the average of words that are not recalled in the immediate and delayed word recall tasks. The memory score ranges from 0 to 10. The orientation test comprises four questions about the day of the week, the month, the date of the month, and the year. One point is given for each wrong answer, and the range is from 0 to 4. | | |
|  |  |  |
|  |  |  |

Table S3 showed extracted variables and missing proportions of variable.

| Variables | ToTal | Total | Number of Missing | Missing proportion |
| --- | --- | --- | --- | --- |
|  |  |  |  |  |
| Age, year | 7995 | 7995 | 0 | 0% |
| Gender, n(%) | 7995 | 7995 | 0 | 0% |
| Diabetes, n(%) | 7995 | 7995 | 0 | 0% |
| Hypertension, n(%) | 7995 | 7995 | 0 | 0% |
| Heart disease, n(%) | 7995 | 7995 | 0 | 0% |
| Stroke, n(%) | 7995 | 7995 | 0 | 0% |
| Dyslipidemia, n(%) | 7995 | 7926 | 69 | 0.86% |
| Drinking, n(%) | 7995 | 7993 | 2 | 0.03% |
| Smoking, n(%) | 7995 | 7994 | 1 | 0.01% |
| BMI | 7995 | 7093 | 902 | 11.28% |
| SBP | 7995 | 7099 | 896 | 11.21% |
| DBP | 7995 | 7098 | 897 | 11.22% |
| FBG | 7995 | 7983 | 12 | 0.15% |
| HbA1c | 7995 | 7937 | 58 | 0.73% |
| TC | 7995 | 7994 | 1 | 0.01% |
| TG | 7995 | 7995 | 0 | 0% |
| HDL C | 7995 | 7995 | 0 | 0% |
| LDL C | 7995 | 7983 | 12 | 0.15% |
| FI | 7995 | 7995 | 0 | 0% |
| AIP | 7995 | 7995 | 0 | 0% |

### Table S4 Baseline characteristics of individuals classified by outcome (wave 2013 to wave 2020).

| Variables | Total (n = 7995) | Without CMM (n = 7248) | CMM (n = 747) | *P* |
| --- | --- | --- | --- | --- |
|  |  |  |  |  |
| Age, year | 58.00 (51.00, 64.00) | 57.00 (51.00, 64.00) | 59.00 (54.00, 64.00) | **<.001** |
| Gender, n(%) |  |  |  | **0.003** |
| Female | 4042 (50.56) | 3626 (50.03) | 416 (55.69) |  |
| Male | 3953 (49.44) | 3622 (49.97) | 331 (44.31) |  |
| Diabetes, n(%) | 375 (4.69) | 257 (3.55) | 118 (15.80) | **<.001** |
| Hypertension, n(%) | 2046 (25.59) | 1655 (22.83) | 391 (52.34) | **<.001** |
| Heart disease, n(%) | 826 (10.33) | 607 (8.37) | 219 (29.32) | **<.001** |
| Stroke, n(%) | 118 (1.48) | 86 (1.19) | 32 (4.28) | **<.001** |
| Dyslipidemia, n(%) | 765 (9.57) | 581 (8.02) | 184 (24.63) | **<.001** |
| Smoking, n(%) | 3246 (40.60) | 2979 (41.10) | 267 (35.74) | **0.005** |
| Drinking, n(%) | 3261 (40.79) | 2973 (41.02) | 288 (38.55) | 0.192 |
| BMI | 23.20 (20.93, 25.78) | 23.05 (20.84, 25.56) | 24.96 (22.33, 27.52) | **<.001** |
| DBP | 74.50 (67.00, 83.00) | 74.00 (66.50, 82.50) | 77.50 (69.00, 85.50) | **<.001** |
| SBP | 126.00 (113.50, 141.00) | 125.50 (113.00, 140.00) | 130.50 (119.00, 147.00) | **<.001** |
| Glucose | 102.06 (94.32, 112.68) | 101.70 (93.96, 111.24) | 111.06 (99.36, 129.33) | **<.001** |
| HbA1c | 5.10 (4.90, 5.40) | 5.10 (4.90, 5.40) | 5.30 (5.00, 5.80) | **<.001** |
| TC | 190.59 (167.40, 215.34) | 189.82 (166.62, 214.18) | 197.17 (174.55, 224.23) | **<.001** |
| TG | 107.08 (75.22, 155.76) | 104.43 (74.34, 152.22) | 128.32 (91.15, 199.57) | **<.001** |
| HDLC | 49.10 (40.21, 59.54) | 49.48 (40.59, 59.92) | 45.23 (36.73, 54.51) | **<.001** |
| LDLC | 114.43 (93.17, 137.63) | 114.05 (93.17, 136.47) | 119.85 (95.68, 145.36) | **<.001** |
| FI | 10.83 (5.36, 17.30) | 10.60 (5.13, 16.96) | 16.41 (10.71, 24.39) | **<.001** |
| AIP | 0.33 (0.12, 0.56) | 0.32 (0.11, 0.55) | 0.47 (0.25, 0.68) | **<.001** |
| AIPFI | 2.87 (0.72, 6.77) | 2.60 (0.62, 6.20) | 6.54 (2.96, 12.70) | **<.001** |

Table S5 Baseline characteristics of individuals classified by outcome (wave 2018 to wave 2020).

| Variables | Total (n = 4483) | Without CMM (n = 4144) | CMM (n = 339) | *P* |
| --- | --- | --- | --- | --- |
|  |  |  |  |  |
| Age, year | 57.00 (51.00, 63.00) | 57.00 (51.00, 63.00) | 60.00 (54.00, 64.00) | **<.001** |
| Gender, n(%) |  |  |  | 0.144 |
| Female | 2249 (50.17) | 2066 (49.86) | 183 (53.98) |  |
| Male | 2234 (49.83) | 2078 (50.14) | 156 (46.02) |  |
| Diabetes, n(%) | 195 (4.35) | 150 (3.62) | 45 (13.27) | **<.001** |
| Hypertension, n(%) | 1127 (25.14) | 950 (22.92) | 177 (52.21) | **<.001** |
| Heart disease, n(%) | 456 (10.17) | 360 (8.69) | 96 (28.32) | **<.001** |
| Strok , n(%) | 52 (1.16) | 42 (1.01) | 10 (2.95) | **0.003** |
| Dyslipidemia, n(%) | 430 (9.59) | 348 (8.40) | 82 (24.19) | **<.001** |
| Smoking, n(%) | 1826 (40.73) | 1691 (40.81) | 135 (39.82) | 0.723 |
| Drinking, n(%) | 1868 (41.67) | 1731 (41.77) | 137 (40.41) | 0.626 |
| BMI | 23.22 (21.03, 25.81) | 23.09 (20.95, 25.62) | 25.15 (22.45, 27.60) | **<.001** |
| SBP | 125.00 (113.00, 139.50) | 124.50 (112.50, 139.00) | 130.50 (117.50, 147.00) | **<.001** |
| DBP | 74.00 (66.50, 82.50) | 74.00 (66.50, 82.50) | 77.00 (69.00, 85.00) | **<.001** |
| FBG | 101.88 (94.32, 111.33) | 101.52 (94.14, 110.16) | 110.34 (99.18, 125.91) | **<.001** |
| HbA1c | 5.10 (4.90, 5.40) | 5.10 (4.90, 5.40) | 5.30 (5.00, 5.70) | **<.001** |
| TC | 190.59 (166.62, 214.56) | 189.43 (166.62, 213.40) | 199.49 (174.55, 224.81) | **<.001** |
| TG | 107.08 (75.22, 155.76) | 105.32 (74.34, 153.10) | 128.32 (94.25, 203.11) | **<.001** |
| HDLC | 49.10 (40.21, 59.54) | 49.48 (40.59, 59.92) | 43.69 (36.53, 52.77) | **<.001** |
| LDLC | 113.66 (92.78, 136.47) | 113.27 (92.40, 135.70) | 120.23 (94.72, 146.52) | **<.001** |
| FI 2011 | 10.60 (5.13, 17.08) | 10.38 (5.02, 16.74) | 14.40 (10.49, 22.99) | **<.001** |
| FI 2015 | 13.73 (7.81, 20.54) | 12.39 (7.70, 20.31) | 19.75 (13.84, 29.30) | **<.001** |
| AIP baseline | 0.33 (0.13, 0.56) | 0.32 (0.12, 0.55) | 0.48 (0.26, 0.69) | **<.001** |
| AIP 2015 | 0.36 (0.19, 0.57) | 0.36 (0.18, 0.56) | 0.45 (0.30, 0.64) | **<.001** |
| AIPFI baseline | 2.77 (0.71, 6.52) | 2.57 (0.63, 6.07) | 6.35 (2.84, 12.70) | **<.001** |
| AIPFI 2015 | 4.22 (1.67, 8.59) | 3.97 (1.54, 8.01) | 8.73 (4.81, 14.15) | **<.001** |
| Cumulative AIPFI | 47.68 ± 51.75 | 44.49 ± 48.25 | 86.65 ± 73.01 | **<.001** |
| Cumulative AIPFI |  |  |  | **<.001** |
| Q1 | 1121 (25.01) | 1095 (26.42) | 26 (7.67) |  |
| Q2 | 1120 (24.98) | 1078 (26.01) | 42 (12.39) |  |
| Q3 | 1121 (25.01) | 1024 (24.71) | 97 (28.61) |  |
| Q4 | 1121 (25.01) | 947 (22.85) | 174 (51.33) |  |

Table S6 Baseline characteristics of participants based on longitudinal changes in AIPFI (Cluster 1 and Cluster 2 group)

| Variables | Total (n = 4483) | Cluster 1 (n = 3534) | Cluster 2 (n = 949) | *P* |
| --- | --- | --- | --- | --- |
|  |  |  |  |  |
| Age, year | 57.00 (51.00, 63.00) | 57.00 (51.00, 63.00) | 59.00 (52.00, 64.00) | **<.001** |
| Gender, n(%) |  |  |  | **<.001** |
| Female | 2249 (50.17) | 1650 (46.69) | 599 (63.12) |  |
| Male | 2234 (49.83) | 1884 (53.31) | 350 (36.88) |  |
| Diabetes, n(%) | 195 (4.35) | 117 (3.31) | 78 (8.22) | **<.001** |
| Hypertension, n(%) | 1127 (25.14) | 677 (19.16) | 450 (47.42) | **<.001** |
| Heart disease, n(%) | 456 (10.17) | 248 (7.02) | 208 (21.92) | **<.001** |
| Stroke, n(%) | 52 (1.16) | 28 (0.79) | 24 (2.53) | **<.001** |
| Dyslipidemia, n(%) | 430 (9.59) | 255 (7.22) | 175 (18.44) | **<.001** |
| Smoking, n(%) | 1826 (40.73) | 1505 (42.59) | 321 (33.83) | **<.001** |
| Drinking, n(%) | 1868 (41.67) | 1538 (43.52) | 330 (34.77) | **<.001** |
| BMI | 23.22 (21.03, 25.81) | 22.88 (20.81, 25.29) | 24.90 (22.38, 27.58) | **<.001** |
| SBP | 125.00 (113.00, 139.50) | 124.00 (112.00, 137.50) | 129.50 (117.00, 146.50) | **<.001** |
| DBP | 74.00 (66.50, 82.50) | 73.50 (66.00, 82.00) | 76.00 (69.00, 85.00) | **<.001** |
| FBG | 101.88 (94.32, 111.33) | 101.16 (93.78, 109.44) | 105.30 (97.02, 119.52) | **<.001** |
| HbA1c | 5.10 (4.90, 5.40) | 5.10 (4.90, 5.40) | 5.20 (4.90, 5.50) | **<.001** |
| TC | 190.59 (166.62, 214.56) | 187.89 (165.46, 212.63) | 198.71 (172.81, 223.84) | **<.001** |
| TG | 107.08 (75.22, 155.76) | 95.58 (69.92, 134.52) | 164.61 (121.25, 251.34) | **<.001** |
| HDLC | 49.10 (40.21, 59.54) | 51.42 (42.91, 61.86) | 40.98 (33.63, 47.94) | **<.001** |
| LDLC | 113.66 (92.78, 136.47) | 113.66 (93.17, 135.31) | 114.82 (90.08, 140.72) | 0.563 |
| FI baseline | 10.60 (5.13, 17.08) | 8.04 (4.69, 13.84) | 19.64 (13.73, 26.34) | **<.001** |
| FI 2015 | 13.73 (7.81, 20.54) | 10.94 (7.37, 17.08) | 23.66 (17.30, 33.04) | **<.001** |
| AIP baseline | 0.33 (0.13, 0.56) | 0.26 (0.08, 0.47) | 0.60 (0.42, 0.84) | **<.001** |
| AIP 2015 | 0.36 (0.19, 0.57) | 0.31 (0.15, 0.49) | 0.60 (0.44, 0.77) | **<.001** |
| AIPFI baseline | 2.77 (0.71, 6.52) | 1.85 (0.39, 3.99) | 11.10 (7.74, 15.65) | **<.001** |
| AIPFI 2015 | 4.22 (1.67, 8.59) | 3.05 (1.14, 5.75) | 13.88 (10.13, 18.46) | **<.001** |

Table S7.Association between AIPFI and CMM incidence after multivariable adjustment (Covariates include medication history)

| Variables | Model1 | |  | Model2 | |  | Model3 | |  | Model4 | |
| --- | --- | --- | --- | --- | --- | --- | --- | --- | --- | --- | --- |
|  | HR (95%CI) | *P* |  | HR (95%CI) | *P* |  | HR (95%CI) | *P* |  | HR (95%CI) | *P* |
| AIPFI | 1.06 (1.06 ~ 1.07) | **<.001** |  | 1.06 (1.05 ~ 1.07) | **<.001** |  | 1.03 (1.02 ~ 1.04) | **<.001** |  | 1.04 (1.02 ~ 1.05) | **<.001** |
| AIPFI |  |  |  |  |  |  |  |  |  |  |  |
| Q1 | 1.00 (Reference) |  |  | 1.00 (Reference) |  |  | 1.00 (Reference) |  |  | 1.00 (Reference) |  |
| Q2 | 1.61 (1.19 ~ 2.17) | **0.002** |  | 1.61 (1.20 ~ 2.17) | **0.002** |  | 1.64 (1.21 ~ 2.22) | **0.001** |  | 1.52 (1.11 ~ 2.09) | **0.009** |
| Q3 | 3.05 (2.33 ~ 4.01) | **<.001** |  | 3.00 (2.28 ~ 3.93) | **<.001** |  | 2.21 (1.67 ~ 2.91) | **<.001** |  | 2.07 (1.52~ 2.82) | **<.001** |
| Q4 | 5.75 (4.45 ~ 7.43) | **<.001** |  | 5.56 (4.30 ~ 7.20) | **<.001** |  | 2.67 (2.04 ~ 3.51) | **<.001** |  | 2.51 (1.80 ~ 3.51) | **<.001** |
| HR: Hazard Ratio, CI: Confidence Interval | | | | | | | | | | | |
| Model1: Crude | | | | | | | | | | | |
| Model2: Adjust: Age,gender | | | | | | | | | | | |
| Model3: Adjust: Age, gender, hypertension, diabetes,dyslipidemia, heart disease, stroke, smoking, drinking, antidiabetic drugs, lipid-lowering drugs, antihypertensive drugs. | | | | | | | | | | | |
| Model4: Adjust: Age, gender, hypertension, diabetes, dyslipidemia, heart disease, stroke, smoking, drinking, antidiabetic drugs, lipid-lowering drugs, antihypertensive drugs, BMI, SBP, DBP, FBG, HbA1c, TC, TG, HDLC, LDLC. | | | | | | | | | | | |

Table S8 Association between AIPFI and CMM incidence after multivariable adjustment in participants who were not receiving treatment for diabetes, hypertension, dyslipidemia, heart disease, or stroke at baseline (N=5,908)

| Variables | Model1 | |  | Model2 | |  | Model3 | |  | Model4 | |
| --- | --- | --- | --- | --- | --- | --- | --- | --- | --- | --- | --- |
|  | HR (95%CI) | *P* |  | HR (95%CI) | *P* |  | HR (95%CI) | *P* |  | HR (95%CI) | *P* |
| AIPFI | 1.05 (1.04 ~ 1.07) | **<.001** |  | 1.05 (1.04 ~ 1.06) | **<.001** |  | 1.03 (1.02 ~ 1.05) | **<.001** |  | 1.03 (1.01 ~ 1.05) | **<.001** |
| AIPFI |  |  |  |  |  |  |  |  |  |  |  |
| Q1 | 1.00 (Reference) |  |  | 1.00 (Reference) |  |  | 1.00 (Reference) |  |  | 1.00 (Reference) |  |
| Q2 | 1.60 (1.04 ~ 2.45) | **0.032** |  | 1.61 (1.05 ~ 2.48) | **0.029** |  | 1.62 (1.05 ~ 2.48) | **0.029** |  | 1.71 (1.08 ~ 2.70) | **0.022** |
| Q3 | 3.13 (2.12 ~ 4.61) | **<.001** |  | 3.13 (2.12 ~ 4.62) | **<.001** |  | 2.76 (1.87 ~ 4.09) | **<.001** |  | 2.90 (1.86 ~ 4.51) | **<.001** |
| Q4 | 4.45 (3.06 ~ 6.47) | **<.001** |  | 4.37 (3.00 ~ 6.36) | **<.001** |  | 3.02 (2.05 ~ 4.44) | **<.001** |  | 3.42 (2.12 ~ 5.52) | **<.001** |
| HR: Hazard Ratio, CI: Confidence Interval | | | | | | | | | | | |
| Model1: Crude | | | | | | | | | | | |
| Model2: Adjust: Age,gender | | | | | | | | | | | |
| Model3: Adjust: Age, gender, hypertension, diabetes,dyslipidemia, heart disease, stroke, smoking, drinking. | | | | | | | | | | | |
| Model4: Adjust: Age, gender, hypertension, diabetes, dyslipidemia, heart disease, stroke, smoking, drinking, BMI, SBP, DBP, FBG, HbA1c, TC, TG, HDLC, LDLC. | | | | | | | | | | | |

Table S9 Sensitivity analysis of the AIPFI on the risks of developing CMM in participants without diabetes, heart disease or stroke at baseline (N=6,676)

| Variables | Model1 | |  | Model2 | |  | Model3 | |  | Model4 | |
| --- | --- | --- | --- | --- | --- | --- | --- | --- | --- | --- | --- |
|  | HR (95%CI) | *P* |  | HR (95%CI) | *P* |  | HR (95%CI) | *P* |  | HR (95%CI) | *P* |
| AIPFI | 1.06 (1.05 ~ 1.07) | **<.001** |  | 1.05 (1.04 ~ 1.07) | **<.001** |  | 1.04 (1.03 ~ 1.05) | **<.001** |  | 1.04 (1.03 ~ 1.06) | **<.001** |
| AIPFI |  |  |  |  |  |  |  |  |  |  |  |
| Q1 | 1.00 (Reference) |  |  | 1.00 (Reference) |  |  | 1.00 (Reference) |  |  | 1.00 (Reference) |  |
| Q2 | 1.97 (1.35 ~ 2.88) | **<.001** |  | 1.98 (1.36 ~ 2.89) | **<.001** |  | 1.93 (1.32 ~ 2.81) | **<.001** |  | 1.81 (1.21 ~ 2.71) | **0.004** |
| Q3 | 3.39 (2.38 ~ 4.84) | **<.001** |  | 3.33 (2.34 ~ 4.76) | **<.001** |  | 2.82 (1.97 ~ 4.04) | **<.001** |  | 2.68 (1.79 ~ 4.02) | **<.001** |
| Q4 | 4.95 (3.50 ~ 7.02) | **<.001** |  | 4.78 (3.36 ~ 6.78) | **<.001** |  | 3.46 (2.41 ~ 4.97) | **<.001** |  | 3.49 (2.25 ~ 5.42) | **<.001** |
| HR: Hazard Ratio, CI: Confidence Interval | | | | | | | | | | | |
| Model1: Crude | | | | | | | | | | | |
| Model2: Adjust: Age, gender. | | | | | | | | | | | |
| Model3: Adjust: Age, gender, smoking, hypertension, dyslipidemia, drinking. | | | | | | | | | | | |
| Model4: Adjust: Age, gender, smoking, hypertension, dyslipidemia, drinking, DBP, SBP, glucose, HbA1c, BMI, TC, TG, HDLC, LDLC. | | | | | | | | | | | |

Table S10 Sensitivity analysis of the AIPFI on the risks of developing diabetes.

| Variables | Model1 | |  | Model2 | |  | Model3 | |  | Model4 | |
| --- | --- | --- | --- | --- | --- | --- | --- | --- | --- | --- | --- |
|  | HR (95%CI) | *P* |  | HR (95%CI) | *P* |  | HR (95%CI) | *P* |  | HR (95%CI) | *P* |
| AIPFI | 1.05 (1.04 ~ 1.05) | **<.001** |  | 1.04 (1.04 ~ 1.05) | **<.001** |  | 1.04 (1.03 ~ 1.05) | **<.001** |  | 1.03 (1.02 ~ 1.04) | **<.001** |
| AIPFI |  |  |  |  |  |  |  |  |  |  |  |
| Q1 | 1.00 (Reference) |  |  | 1.00 (Reference) |  |  | 1.00 (Reference) |  |  | 1.00 (Reference) |  |
| Q2 | 1.59 (1.27 ~ 1.99) | **<.001** |  | 1.58 (1.27 ~ 1.98) | **<.001** |  | 1.57 (1.25 ~ 1.96) | **<.001** |  | 1.13 (0.88 ~ 1.47) | 0.336 |
| Q3 | 2.32 (1.88 ~ 2.86) | **<.001** |  | 2.30 (1.86 ~ 2.84) | **<.001** |  | 2.13 (1.72 ~ 2.63) | **<.001** |  | 1.52 (1.18 ~ 1.96) | **0.001** |
| Q4 | 3.58 (2.93 ~ 4.38) | **<.001** |  | 3.52 (2.88 ~ 4.32) | **<.001** |  | 2.97 (2.40 ~ 3.67) | **<.001** |  | 1.97 (1.49 ~ 2.60) | **<.001** |
| HR: Hazard Ratio, CI: Confidence Interval | | | | | | | | | | | |
| Model1: Crude | | | | | | | | | | | |
| Model2: Adjust: Age, gender. | | | | | | | | | | | |
| Model3: Adjust: Age, gender, hypertension, heart disease,dyslipidemia, stroke, smoking, drinking. | | | | | | | | | | | |
| Model4: Adjust: Age, gender, hypertension, heart disease, dyslipidemia, stroke, smoking, drinking, BMI, SBP, DBP, FBG, HbA1c, TC, TG, HDLC, LDLC. | | | | | | | | | | | |

Table S11 Sensitivity analysis of the AIPFI on the risks of developing stroke.

| Variables | Model1 | |  | Model2 | |  | Model3 | |  | Model4 | |
| --- | --- | --- | --- | --- | --- | --- | --- | --- | --- | --- | --- |
|  | HR (95%CI) | *P* |  | HR (95%CI) | *P* |  | HR (95%CI) | *P* |  | HR (95%CI) | *P* |
| AIPFI | 1.05 (1.04 ~ 1.05) | **<.001** |  | 1.04 (1.04 ~ 1.05) | **<.001** |  | 1.03 (1.02 ~ 1.04) | **<.001** |  | 1.03 (1.02 ~ 1.05) | **<.001** |
| AIPFI |  |  |  |  |  |  |  |  |  |  |  |
| Q1 | 1.00 (Reference) |  |  | 1.00 (Reference) |  |  | 1.00 (Reference) |  |  | 1.00 (Reference) |  |
| Q2 | 1.55 (1.19 ~ 2.03) | **0.001** |  | 1.56 (1.20 ~ 2.04) | **0.001** |  | 1.52 (1.16 ~ 1.98) | **0.002** |  | 1.77 (1.30 ~ 2.42) | **<.001** |
| Q3 | 2.34 (1.82 ~ 3.00) | **<.001** |  | 2.34 (1.82 ~ 3.00) | **<.001** |  | 1.99 (1.54 ~ 2.56) | **<.001** |  | 2.30 (1.68 ~ 3.17) | **<.001** |
| Q4 | 3.12 (2.45 ~ 3.97) | **<.001** |  | 3.09 (2.42 ~ 3.94) | **<.001** |  | 2.05 (1.58 ~ 2.65) | **<.001** |  | 2.37 (1.66 ~ 3.39) | **<.001** |
| HR: Hazard Ratio, CI: Confidence Interval | | | | | | | | | | | |
| Model1: Crude | | | | | | | | | | | |
| Model2: Adjust: Age, gender. | | | | | | | | | | | |
| Model3: Adjust: Age, gender, hypertension, diabetes,dyslipidemia, heart disease, smoking, drinking. | | | | | | | | | | | |
| Model4: Adjust: Age, gender, hypertension, diabetes, dyslipidemia, heart disease, smoking, drinking, BMI, SBP, DBP, FBG, HbA1c, TC, TG, HDLC, LDLC. | | | | | | | | | | | |

Table S12 Sensitivity analysis of the AIPFI on the risks of developing heart disease.

| Variables | Model1 | |  | Model2 | |  | Model3 | |  | Model4 | |
| --- | --- | --- | --- | --- | --- | --- | --- | --- | --- | --- | --- |
|  | HR (95%CI) | *P* |  | HR (95%CI) | *P* |  | HR (95%CI) | *P* |  | HR (95%CI) | *P* |
| AIPFI | 1.03 (1.03 ~ 1.04) | **<.001** |  | 1.03 (1.02 ~ 1.04) | **<.001** |  | 1.02 (1.01 ~ 1.03) | **<.001** |  | 1.03 (1.02 ~ 1.05) | **<.001** |
| AIPFI |  |  |  |  |  |  |  |  |  |  |  |
| Q1 | 1.00 (Reference) |  |  | 1.00 (Reference) |  |  | 1.00 (Reference) |  |  | 1.00 (Reference) |  |
| Q2 | 1.11 (0.94 ~ 1.32) | 0.233 |  | 1.11 (0.93 ~ 1.31) | 0.242 |  | 1.11 (0.94 ~ 1.32) | 0.217 |  | 1.18 (0.97 ~ 1.44) | 0.101 |
| Q3 | 1.38 (1.17 ~ 1.62) | **<.001** |  | 1.33 (1.13 ~ 1.57) | **<.001** |  | 1.23 (1.04 ~ 1.46) | **0.014** |  | 1.45 (1.18 ~ 1.78) | **<.001** |
| Q4 | 1.99 (1.70 ~ 2.32) | **<.001** |  | 1.86 (1.59 ~ 2.17) | **<.001** |  | 1.52 (1.29 ~ 1.80) | **<.001** |  | 1.90 (1.51 ~ 2.40) | **<.001** |
| HR: Hazard Ratio, CI: Confidence Interval | | | | | | | | | | | |
| Model1: Crude | | | | | | | | | | | |
| Model2: Adjust: Age, gender. | | | | | | | | | | | |
| Model3: Adjust: Age, gender, hypertension, diabetes,dyslipidemia, stroke, smoking, drinking. | | | | | | | | | | | |
| Model4: Adjust: Age, gender, hypertension, diabetes, dyslipidemia, stroke, smoking, drinking, BMI, SBP, DBP, FBG, HbA1c, TC, TG, HDLC, LDLC. | | | | | | | | | | | |

Table S13 Association between AIPFI and CMM incidence after multivariable adjustment (Using data from Wave 3 as the baseline).

| Variables | Model1 | |  | Model2 | |  | Model3 | |  | Model4 | |
| --- | --- | --- | --- | --- | --- | --- | --- | --- | --- | --- | --- |
|  | HR (95%CI) | *P* |  | HR (95%CI) | *P* |  | HR (95%CI) | *P* |  | HR (95%CI) | *P* |
| AIPFI 2015 | 1.06 (1.05 ~ 1.06) | **<.001** |  | 1.06 (1.05 ~ 1.06) | **<.001** |  | 1.02 (1.02 ~ 1.03) | **<.001** |  | 1.02 (1.01 ~ 1.04) | **<.001** |
| AIPFI 2015 |  | | | | | | | | | | |
| Q1 | 1.00 (Reference) |  |  | 1.00 (Reference) |  |  | 1.00 (Reference) |  |  | 1.00 (Reference) |  |
| Q2 | 1.91 (1.37 ~ 2.67) | **<.001** |  | 1.90 (1.36 ~ 2.65) | **<.001** |  | 1.54 (1.10 ~ 2.15) | **0.012** |  | 1.61 (1.13 ~ 2.29) | **0.008** |
| Q3 | 3.23 (2.37 ~ 4.40) | **<.001** |  | 3.17 (2.32 ~ 4.32) | **<.001** |  | 1.78 (1.30 ~ 2.45) | **<.001** |  | 1.95 (1.37 ~ 2.77) | **<.001** |
| Q4 | 6.91 (5.17 ~ 9.23) | **<.001** |  | 6.52 (4.87 ~ 8.73) | **<.001** |  | 2.50 (1.84 ~ 3.39) | **<.001** |  | 2.83 (1.95 ~ 4.12) | **<.001** |
| HR: Hazard Ratio, CI: Confidence Interval | | | | | | | | | | | |
| Model1: Crude | | | | | | | | | | | |
| Model2: Adjust: Age,gender. | | | | | | | | | | | |
| Model3: Adjust: Age, Gender, Hypertension, Diabetes, Dyslipidemia, Heart disease, Stroke. | | | | | | | | | | | |
| Model4: Adjust: Age, Gender, Hypertension, Diabetes, Dyslipidemia, Heart disease, Stroke, Age, BMI, SBP, DBP, FBG, HbA1c, TC, TG, HDLC, LDLC. | | | | | | | | | | | |

Table S14 Sensitivity analysis of the AIPFI on the risks of developing CMM (Standardization, Stratification by sign, and Outlier removal)

| Variables | Model1 | |  | Model2 | |  | Model3 | |  | Model4 | |
| --- | --- | --- | --- | --- | --- | --- | --- | --- | --- | --- | --- |
|  | HR (95%CI) | *P* |  | HR (95%CI) | *P* |  | HR (95%CI) | *P* |  | HR (95%CI) | *P* |
| AIPFI  Z score | 1.48 (1.43 ~ 1.54) | **<.001** |  | 1.46 (1.41 ~ 1.52) | **<.001** |  | 1.23 (1.17 ~ 1.30) | **<.001** |  | 1.27 (1.18 ~ 1.36) | **<.001** |
| AIPFI > 0 | 1.06 (1.05 ~ 1.07) | **<.001** |  | 1.06 (1.05 ~ 1.06) | **<.001** |  | 1.03 (1.02 ~ 1.04) | **<.001** |  | 1.04 (1.03 ~ 1.05) | **<.001** |
| AIPFI ≤ 0 | 0.84 (0.77 ~ 0.92) | **<.001** |  | 0.84 (0.77 ~ 0.92) | **<.001** |  | 0.85 (0.78 ~ 0.93) | **<.001** |  | 0.79(0.70 ~ 0.92) | **0.001** |
| AIPFI_outlier removal | 1.07 (1.06 ~ 1.08) | **<.001** |  | 1.07 (1.06 ~ 1.07) | **<.001** |  | 1.03 (1.03 ~ 1.04) | **<.001** |  | 1.04 (1.03 ~ 1.05) | **<.001** |
| HR: Hazard Ratio, CI: Confidence Interval; Outlier removal:Outlier removal method: For continuous variables with a skewed distribution, values above the 99.9th percentile were set to the 99.9th percentile, and values below the 0.1st percentile were set to the 0.1st percentile. | | | | | | | | | | | |
| Model1: Crude | | | | | | | | | | | |
| Model2: Adjust: Age, gender. | | | | | | | | | | | |
| Model3: Adjust: Age, gender, hypertension, heart disease,dyslipidemia, stroke, smoking, drinking. | | | | | | | | | | | |
| Model4: Adjust: Age, gender, hypertension, heart disease, dyslipidemia, stroke, smoking, drinking, BMI, SBP, DBP, FBG, HbA1c, TC, TG, HDLC, LDLC. | | | | | | | | | | | |

Table S15. Sensitivity analysis of the association between AIPFI and CMM after reclassifying smoking and drinking status as “Current/Former/Never”

| Variables | Model1 | |  | Model2 | |  | Model3 | |  | Model4 | |
| --- | --- | --- | --- | --- | --- | --- | --- | --- | --- | --- | --- |
|  | HR (95%CI) | *P* |  | HR (95%CI) | *P* |  | HR (95%CI) | *P* |  | HR (95%CI) | *P* |
| AIPFI | 1.06 (1.06 ~ 1.07) | **<.001** |  | 1.06 (1.05 ~ 1.07) | **<.001** |  | 1.03 (1.02 ~ 1.04) | **<.001** |  | 1.03 (1.02 ~ 1.05) | **<.001** |
| AIPFI four |  |  |  |  |  |  |  |  |  |  |  |
| Q1 | 1.00 (Reference) |  |  | 1.00 (Reference) |  |  | 1.00 (Reference) |  |  | 1.00 (Reference) |  |
| Q2 | 1.61 (1.19 ~ 2.17) | **0.002** |  | 1.61 (1.20 ~ 2.17) | **0.002** |  | 1.62 (1.20 ~ 2.19) | **0.002** |  | 1.49 (1.09 ~ 2.05) | **0.013** |
| Q3 | 3.05 (2.33 ~ 4.01) | **<.001** |  | 3.00 (2.28 ~ 3.93) | **<.001** |  | 2.18 (1.65 ~ 2.87) | **<.001** |  | 2.01 (1.48 ~ 2.74) | **<.001** |
| Q4 | 5.75 (4.45 ~ 7.43) | **<.001** |  | 5.56 (4.30 ~ 7.20) | **<.001** |  | 2.65 (2.02 ~ 3.48) | **<.001** |  | 2.40 (1.71 ~ 3.36) | **<.001** |
| HR: Hazard Ratio, CI: Confidence Interval | | | | | | | | | | | |
| Model1: Crude | | | | | | | | | | | |
| Model2: Adjust: Age, gender. | | | | | | | | | | | |
| Model3: Adjust: Age, gender, hypertension, heart disease,dyslipidemia, stroke, smoking, drinking. | | | | | | | | | | | |
| Model4: Adjust: Age, gender, hypertension, heart disease, dyslipidemia, stroke, smoking, drinking, BMI, SBP, DBP, FBG, HbA1c, TC, TG, HDLC, LDLC. | | | | | | | | | | | |

Table S16. Sensitivity analysis: IPW-weighted Cox regression analysis for the association between AIPFI and CMM

| model | exposure | n | events | HR | P value | 95% CI |
| --- | --- | --- | --- | --- | --- | --- |
| IPW-weighted Cox | AIPFI (high vs low) | 7995 | 747 | 1.72 | 4.307e-05 | 1.33-2.23 |

Table S17 Sensitivity analysis of the relationship between AIPFI and CMM using the Fine and Gray proportional subdistribution hazards model.

| model | sHR | 95% CI | *P* |
| --- | --- | --- | --- |
| AIPFI Q1 | Ref | | |
| Q2 | 1.56 | 1.14-2.13 | 0.005 |
| Q3 | 2.12 | 1.56-2.89 | <.001 |
| Q4 | 2.55 | 1.83-3.57 | <.001 |

CI = confidence interval; sHR= subdistribution hazard ratio. Adjust: Age, Gender, Hypertension, Diabetes, Dyslipidemia, Heart disease, Stroke, Smoking, Drinking, Age, BMI, SBP, DBP, FBG, HbA1c, TC, TG, HDLC, LDLC.

Table S18 Collinearity analysis using variance inflation factor (VIF).

| Variables | VIF |
| --- | --- |
| TC | 10.350 |
| LDLC | 8.324 |
| TG | 4.415 |
| HDLC | 2.726 |
| SBP | 2.634 |
| DBP | 2.471 |
| Gender | 2.280 |
| Smoking | 2.012 |
| Glucose | 1.986 |
| AIPFI | 1.947 |
| HbA1c | 1.918 |
| Drinking | 1.457 |
| Hypertension | 1.313 |
| BMI | 1.310 |
| Age | 1.285 |
| Diabetes | 1.277 |
| Dyslipidemia | 1.112 |
| Heart_disease | 1.102 |
| Stroke | 1.031 |

Table S19 Sensitivity analysis of the AIPFI on the risks of developing CMM after excluding variables with VIF >5 and TG.

| Variables | Model1 | |  | Model2 | |  | Model3 | |  | Model4 | |
| --- | --- | --- | --- | --- | --- | --- | --- | --- | --- | --- | --- |
|  | HR (95%CI) | *P* |  | HR (95%CI) | *P* |  | HR (95%CI) | *P* |  | HR (95%CI) | *P* |
| AIPFI | 1.06 (1.06 ~ 1.07) | **<.001** |  | 1.06 (1.05 ~ 1.07) | **<.001** |  | 1.03 (1.02 ~ 1.04) | **<.001** |  | 1.02 (1.02 ~ 1.03) | **<.001** |
| AIPFI |  |  |  |  |  |  |  |  |  |  |  |
| Q1 | 1.00 (Reference) |  |  | 1.00 (Reference) |  |  | 1.00 (Reference) |  |  | 1.00 (Reference) |  |
| Q2 | 1.61 (1.19 ~ 2.17) | **0.002** |  | 1.61 (1.20 ~ 2.17) | **0.002** |  | 1.60 (1.18 ~ 2.16) | **0.002** |  | 1.46 (1.08 ~ 1.98) | **0.013** |
| Q3 | 3.05 (2.33 ~ 4.01) | **<.001** |  | 3.00 (2.28 ~ 3.93) | **<.001** |  | 2.16 (1.64 ~ 2.84) | **<.001** |  | 1.94 (1.47 ~ 2.56) | **<.001** |
| Q4 | 5.75 (4.45 ~ 7.43) | **<.001** |  | 5.56 (4.30 ~ 7.20) | **<.001** |  | 2.63 (2.00 ~ 3.44) | **<.001** |  | 2.20 (1.67 ~ 2.90) | **<.001** |
| HR: Hazard Ratio, CI: Confidence Interval | | | | | | | | | | | |
| Model1: Crude | | | | | | | | | | | |
| Model2: Adjust:Age, gender. | | | | | | | | | | | |
| Model3: Adjust: Age, gender, hypertension, heart disease,dyslipidemia, stroke, smoking, drinking. | | | | | | | | | | | |
| Model4: Adjust: Age, gender, hypertension, heart disease, dyslipidemia, stroke, smoking, drinking, BMI, SBP, DBP, FBG, HbA1c. | | | | | | | | | | | |

Table S20 Cox regression results for the associations of longitudinal AIPFI change patterns (from 2012 to 2015 based on three clusters) with CMM risk.

| Variables | Model1 | |  | Model2 | |  | Model3 | |  | Model4 | |
| --- | --- | --- | --- | --- | --- | --- | --- | --- | --- | --- | --- |
|  | HR (95%CI) | *P* |  | HR (95%CI) | *P* |  | HR (95%CI) | *P* |  | HR (95%CI) | *P* |
| Cluster 1 | 1.00 (Reference) |  |  | 1.00 (Reference) |  |  | 1.00 (Reference) |  |  | 1.00 (Reference) |  |
| Cluster 2 | 3.30 (2.58 ~ 4.22) | **<.001** |  | 3.30 (2.58 ~ 4.23) | **<.001** |  | 2.19 (1.69 ~ 2.84) | **<.001** |  | 1.99 (1.53 ~ 2.59) | **<.001** |
| Cluster 3 | 6.81 (5.03 ~ 9.20) | **<.001** |  | 6.63 (4.87 ~ 9.04) | **<.001** |  | 2.93 (2.07 ~ 4.14) | **<.001** |  | 2.44 (1.71 ~ 3.48) | **<.001** |
| HR: Hazard Ratio, CI: Confidence Interval | | | | | | | | | | | |
| Model1: Crude | | | | | | | | | | | |
| Model2: Adjust:Age, gender. | | | | | | | | | | | |
| Model3: Adjust: Age, gender, hypertension, heart disease,dyslipidemia, stroke, smoking, drinking. | | | | | | | | | | | |
| Model4: Adjust: Age, gender, hypertension, heart disease, dyslipidemia, stroke, smoking, drinking, BMI, SBP, DBP, FBG, HbA1c. | | | | | | | | | | | |

Table S21 **Proportional Hazards Assumption Test (Schoenfeld Residuals Method)**

| Variables | P-value | P-value after stratification by Diabetes, Hypertension, and Heart_disease |
| --- | --- | --- |
| Age | 0.960 | 0.50 |
| Gender | 0.518 | 0.73 |
| Diabetes | 0.001 | / |
| Hypertension | 0.004 | / |
| Heart disease | 0.006 | / |
| Stroke | 0.125 | / |
| Dyslipidemia | 0.666 | 0.22 |
| Drinking | 0.612 | 0.82 |
| Smoking | 0.769 | 0.67 |
| BMI | 0.055 | 0.50 |
| SBP | 0.604 | 0.69 |
| DBP | 0.740 | 0.78 |
| Glucose | 0.447 | 0.38 |
| HbA1c | 0.503 | 0.21 |
| TC | 0.196 | 0.08 |
| TG | 0.850 | 0.46 |
| HDL C | 0.888 | 0.39 |
| LDL C | 0.395 | 0.28 |
| AIPFI | 0.574 | 0.17 |
| GlOBAL | <0.001 | 0.63 |

Table S22 Multivariable Cox regression results.

| Variables | HR(95%CI) | P-value |
| --- | --- | --- |
| Age | 1.01 (1.00–1.02) | 0.08 |
| Gender | 1.03 (0.83–1.27) | 0.82 |
| Smoking | 0.94 (0.77–1.15) | 0.53 |
| BMI | 1.04 (1.02–1.06) | <0.001 |
| Dyslipidemia | 1.68 (1.40–2.02) | <0.001 |
| DBP | 1.00 (0.99–1.01) | 0.67 |
| SBP | 1.00 (1.00–1.01) | 0.18 |
| Glucose | 1.00 (1.00–1.01) | <0.001 |
| HbA1c | 1.16 (1.05–1.28) | <0.001 |
| TC | 1.00 (1.00–1.01) | 0.81 |
| TG | 1.00 (1.00–1.00) | 0.02 |
| HDLC | 1.00 (0.99–1.00) | 0.46 |
| LDLC | 1.00 (1.00–1.01) | 0.51 |
| Drinking | 1.08 (0.90–1.29) | 0.42 |
| AIPFI | 1.04 (1.03–1.05) | <0.001 |

Note:Stratification variables in the model were Diabetes, Hypertension, Heart_disease, and Stroke. HR: hazard ratio; CI: confidence interval.

Table S23.Incremental predictive value of indicators derived from NRI and IDI

| **Indicator** | **NRI（95%CI）** | **P** | **IDI（95%CI）** | **P** |
| --- | --- | --- | --- | --- |
| Baseline | Reference | Reference | Reference | Reference |
| Baseline+AIPFI | 0.338(0.244 - 0.431) | <0.001 | 0.013(0.0061 - 0.02) | <0.001 |

Table S24 Baseline characteristics of participants randomly divided by a 7:3 ratio into a training cohort and a validation cohort.

| Variables | Total (n = 7995) | test (n = 2399) | train (n = 5596) | *P* |
| --- | --- | --- | --- | --- |
|  |  |  |  |  |
| Age | 58.00 (51.00, 64.00) | 58.00 (51.00, 64.00) | 58.00 (51.00, 64.00) | 0.825 |
| Gender, n(%) |  |  |  | 0.499 |
| Female | 4042 (50.56) | 1199 (49.98) | 2843 (50.80) |  |
| Male | 3953 (49.44) | 1200 (50.02) | 2753 (49.20) |  |
| Diabetes, n(%) | 375 (4.69) | 111 (4.63) | 264 (4.72) | 0.860 |
| Hypertension, n(%) | 2046 (25.59) | 601 (25.05) | 1445 (25.82) | 0.470 |
| Heart disease, n(%) | 826 (10.33) | 269 (11.21) | 557 (9.95) | 0.090 |
| Stroke, n(%) | 118 (1.48) | 39 (1.63) | 79 (1.41) | 0.467 |
| Dyslipidemia, n(%) | 765 (9.57) | 219 (9.13) | 546 (9.76) | 0.382 |
| Smoking, n(%) | 3246 (40.60) | 995 (41.48) | 2251 (40.23) | 0.297 |
| Drinking, n(%) | 3261 (40.79) | 999 (41.64) | 2262 (40.42) | 0.309 |
| BMI | 23.20 (20.93, 25.78) | 23.11 (20.82, 25.76) | 23.23 (20.99, 25.79) | 0.311 |
| SBP | 126.00 (113.50, 141.00) | 125.50 (113.00, 140.50) | 126.00 (114.00, 141.00) | 0.140 |
| DBP | 74.50 (67.00, 83.00) | 74.00 (66.50, 83.00) | 74.50 (67.00, 83.00) | 0.191 |
| Glucose | 102.06 (94.32, 112.68) | 102.06 (94.32, 113.04) | 102.24 (94.14, 112.50) | 0.662 |
| HbA1c | 5.10 (4.90, 5.40) | 5.10 (4.90, 5.40) | 5.10 (4.90, 5.40) | 0.783 |
| TC | 190.59 (167.40, 215.34) | 190.21 (165.85, 215.34) | 190.98 (167.78, 215.34) | 0.248 |
| TG | 107.08 (75.22, 155.76) | 107.08 (77.00, 157.53) | 106.20 (74.34, 155.76) | 0.175 |
| HDLC | 49.10 (40.21, 59.54) | 49.10 (39.82, 59.15) | 49.10 (40.21, 59.63) | 0.362 |
| LDLC | 114.43 (93.17, 137.63) | 113.27 (91.62, 136.08) | 115.21 (94.33, 138.02) | **0.024** |
| AIPFI | 2.87 (0.72, 6.77) | 2.96 (0.78, 6.81) | 2.85 (0.69, 6.76) | 0.291 |

Table S25 Baseline characteristics of participants between the CMM and non-CMM groups into a training cohort.

| Variables | Total (n = 5596) | Without CMM (n = 5070) | CMM (n = 526) | *P* |
| --- | --- | --- | --- | --- |
|  |  |  |  |  |
| Age | 58.00 (51.00, 64.00) | 57.00 (51.00, 64.00) | 59.50 (54.00, 64.75) | **<.001** |
| Gender |  |  |  | **0.005** |
| Female | 2843 (50.80) | 2545 (50.20) | 298 (56.65) |  |
| Male | 2753 (49.20) | 2525 (49.80) | 228 (43.35) |  |
| Diabetes | 264 (4.72) | 178 (3.51) | 86 (16.35) | **<.001** |
| Hypertension | 1445 (25.82) | 1168 (23.04) | 277 (52.66) | **<.001** |
| Heart disease | 557 (9.95) | 404 (7.97) | 153 (29.09) | **<.001** |
| Stroke, n(%) | 79 (1.41) | 60 (1.18) | 19 (3.61) | **<.001** |
| Dyslipidemia, | 546 (9.76) | 416 (8.21) | 130 (24.71) | **<.001** |
| Smoking | 2251 (40.23) | 2057 (40.57) | 194 (36.88) | 0.100 |
| Drinking | 2262 (40.42) | 2060 (40.63) | 202 (38.40) | 0.322 |
| BMI | 23.23 (20.99, 25.79) | 23.08 (20.89, 25.58) | 24.84 (22.31, 27.37) | **<.001** |
| SBP | 126.00 (114.00, 141.00) | 125.50 (113.12, 140.00) | 130.50 (119.50, 146.50) | **<.001** |
| DBP | 74.50 (67.00, 83.00) | 74.50 (67.00, 82.50) | 77.75 (69.50, 85.50) | **<.001** |
| FBG | 102.24 (94.14, 112.50) | 101.70 (93.96, 111.24) | 111.60 (99.05, 132.03) | **<.001** |
| HbA1c | 5.10 (4.90, 5.40) | 5.10 (4.90, 5.40) | 5.30 (5.00, 5.80) | **<.001** |
| TC | 190.98 (167.78, 215.34) | 190.21 (167.40, 214.18) | 198.52 (174.45, 225.19) | **<.001** |
| TG | 106.20 (74.34, 155.76) | 104.43 (73.46, 152.22) | 127.44 (90.27, 200.67) | **<.001** |
| HDLC | 49.10 (40.21, 59.63) | 49.48 (40.59, 60.21) | 45.23 (36.73, 54.80) | **<.001** |
| LDLC | 115.21 (94.33, 138.02) | 114.82 (94.33, 136.86) | 120.23 (95.59, 147.10) | **<.001** |
| AIPFI | 2.85 (0.69, 6.76) | 2.59 (0.60, 6.19) | 6.60 (2.97, 12.41) | **<.001** |

**Fig.S1.** Sensitivity analysis: Cumulative incidence of CMM across AIPFI based on the Fine‑Gray competing risks model.

**Fig.S2.** Sensitivity analysis of the association between changes in AIPFI from 2012 to 2015 and CMM risk, stratified by Clusters = 3.Changes in AIPFI classified into three clusters using the K-means algorithm (A); Cluster 1 (Red)-Stable Low-Risk Group; Cluster 2(Blue)-Persistent moderate-risk group; Cluster 3 (Orange) -Persistent High-Risk Group (B); Mean AIPFI value for the three clusters in 2012 and 2015 (C).

**Fig.S3.** Multivariable Cox regression results (Forest plot of HRs).

**Fig.S4.** ROC analysis of the AIP, FI, and AIPFI. The area under the curve (AUC) for the AIPFI was 0.693, that for the AIP was 0.621, and that for the FI was 0.687. The AIPFI demonstrated the highest predictive value among the three biomarkers.

**Figure S5:** The clinical prediction analysis based on multivariate cox models.The calibration curves in both cohorts (Fig. S5A and S5B). Decision curve analysis revealed in both cohorts (Fig. S5C and S5D).
